# Clinicopathological characteristics of patients with inoperable non‐small cell lung cancer harboring circulating NRF2 pathway mutations

**DOI:** 10.1002/path.70043

**Published:** 2026-03-02

**Authors:** Jouni Härkönen, Satu Tiainen, Jouni Kujala, Linnea Muhonen, Ponnuswamy Mohanasundaram, Tuomas Tikkanen, Ina Pöhner, Tommi Patinen, Simone Adinolfi, Juha P Väyrynen, Päivi Auvinen, Arto Mannermaa, Petri Pölönen, Tuomas Rauramaa, Anna‐Liisa Levonen

**Affiliations:** ^1^ Faculty of Health Sciences, A.I. Virtanen Institute for Molecular Sciences University of Eastern Finland Kuopio Finland; ^2^ Department of Pathology Hospital Nova, Wellbeing Services County of Central Finland Jyväskylä Finland; ^3^ Cancer Center, Kuopio University Hospital, the Wellbeing Services County of North Savo Kuopio Finland; ^4^ Institute of Clinical Medicine University of Eastern Finland Kuopio Finland; ^5^ FICAN East, Kuopio University Hospital, the Wellbeing Services County of North Savo Kuopio Finland; ^6^ Institute of Clinical Medicine, Clinical Pathology and Forensic Medicine University of Eastern Finland Kuopio Finland; ^7^ Department of Biological and Environmental Science University of Jyväskylä Jyväskylä Finland; ^8^ School of Pharmacy, Faculty of Health Sciences University of Eastern Finland Kuopio Finland; ^9^ Translational Medicine Research Unit, University of Oulu, Oulu University Hospital, and Medical Research Center Oulu Oulu Finland; ^10^ Biobank of Eastern Finland, Kuopio University Hospital Kuopio Finland; ^11^ Multidisciplinary Cancer Research Community University of Eastern Finland Kuopio Finland; ^12^ Department of Pathology St Jude Children's Research Hospital Memphis TN USA; ^13^ Department of Clinical Pathology Kuopio University Hospital Kuopio Finland

**Keywords:** NFE2L2, KEAP1, CUL3, AKR1B10, AKR1C1, non‐small cell lung cancer, cell‐free DNA, circulating tumor DNA, tumor microenvironment, SMARCA4

## Abstract

Lung cancer is the leading cause of global cancer‐related morbidity and mortality, with tobacco smoking as its strongest risk factor. Nuclear factor erythroid 2‐related factor 2 (NRF2) is a redox‐regulated transcription factor frequently dysregulated in non‐small cell lung cancer (NSCLC), leading to aggressive disease and resistance to therapy. In this study, we analyzed circulating cell‐free tumor DNA from a real‐world cohort to characterize clinicopathological features and identify risk factors associated with oncogenic NRF2 activation in inoperable NSCLC. Key findings were further validated using retrospective datasets. Our results demonstrate that NRF2 pathway‐mutated NSCLC represents a smoking‐associated, high‐risk molecular subtype frequently accompanied by detrimental SMARCA4 mutations. Importantly, these co‐occurring mutations cumulatively worsen clinical outcomes independently of other risk factors. We show that NRF2‐mutated tumors generally exhibit lower leukocyte infiltration, while high tumor mutation burden independently correlates with increased cytotoxic T lymphocyte density, regardless of NRF2 status. Furthermore, our data indicate that NRF2 activation can be reliably identified through immunohistochemical detection of protein expression of markers AKR1B10 and AKR1C1, both of which correlate with inferior outcomes. As mutations in NRF2‐regulating tumor suppressors *KEAP1* and *CUL3* are not confined to specific hotspot regions, our findings advocate for a multimodal profiling approach combining somatic mutation assessment with protein or transcriptomic evaluation of NRF2 targets. This comprehensive strategy effectively identifies oncogenic NRF2 hyperactivity, enhancing diagnostic accuracy and clinical decision‐making in NSCLC management. © 2026 The Author(s). *The Journal of Pathology* published by John Wiley & Sons Ltd on behalf of The Pathological Society of Great Britain and Ireland.

## Introduction

Lung cancer is the most common malignancy and the leading cause of cancer‐related deaths worldwide [1]. Non‐small cell lung cancer (NSCLC) is the predominant subtype, with smoking as its most important risk factor [2]. While complete surgical resection offers the best outcome, only 15–25% of patients present with operable disease; the majority require radiotherapy, chemotherapy, immunotherapy, and/or targeted therapies [3, 4]. The growing number of targeted options has elevated the role of molecular diagnostics, with next‐generation sequencing (NGS) now a cornerstone of NSCLC profiling [5, 6]. Beyond detecting currently targetable aberrations, NGS aids in identifying groups that do not respond to existing regimens and require novel, tailored interventions [7]. For inoperable NSCLC, core needle biopsies are often used for diagnostic evaluation. However, limited tissue can constrain comprehensive testing, especially as DNA, RNA, and immunohistochemistry (IHC) are increasingly used in parallel [8, 9] Blood‐based circulating tumor DNA (ctDNA) provides a minimally invasive alternative for somatic profiling, enabling serial molecular characterization throughout the course of the disease, with minimal complication risks [10].

Nuclear factor erythroid 2‐related factor 2 (NRF2) is a redox‐responsive transcription factor that maintains intracellular redox homeostasis. The mechanism is based on rapid turnover of NRF2, where its negative regulator, KEAP1, acts as the redox‐responsive element. In basal conditions, KEAP1 serves as a substrate adaptor for CUL3 E3‐ubiquitin ligase, directing NRF2 for ubiquitination and proteasomal degradation via 26S proteasome [11]. Under oxidative or electrophilic stress, the critical cysteine residues of KEAP1 are modified, resulting in conformational changes in KEAP1 and inhibition of the proteasomal degradation machinery. This leads to accumulation and nuclear translocation of NRF2 and, consequently, transcription of a battery of genes, such as enzymes involved in the metabolism of reactive oxygen species and xenobiotics [11, 12].

In cancer, NRF2 is often activated via somatic mutations of *NFE2L2* and *KEAP1*, most remarkably so in NSCLC, in which the combined occurrence of these aberrations has been documented at 15–30% [13, 14, 15]. Oncogenic mutations in *NFE2L2* and *KEAP1* are characterized as gain‐of‐function and loss‐of‐function (LOF), respectively. Thus, *NFE2L2* mutations are typically found as hotspots in its KEAP1‐binding ETGE and DLG domains, while deleterious *KEAP1* mutations are widely dispersed but also include known hotspots [11]. In addition to *NFE2L2* and *KEAP1*, somatic LOF mutations in *CUL3* may lead to NRF2 activation, as *CUL3* has an important functional role in the E3 ligase protein complex. The downstream transcriptional effects of NRF2 extend beyond redox balance, including upregulation of drug‐metabolizing genes and efflux transporters, contributing to therapeutic resistance [16, 17]. Consequently, NRF2 activation has been increasingly recognized as a key mechanism of intrinsic and acquired resistance to a broad spectrum of chemotherapeutic agents and targeted therapies. Despite its clinical significance, NRF2‐driven cancers lack targeted treatments [11, 14].

In this study, we performed multi‐timepoint ctDNA sequencing of 75 lung cancer‐associated genes in a prospective cohort of 73 patients with inoperable NSCLC, focusing on the clinicopathological features of tumors harboring NRF2 pathway activation. We further assessed NRF2 activity through complementary markers and analyzed immune infiltration patterns in matched biopsies. Finally, we validated key findings in external public datasets to provide a comprehensive view of NRF2‐hyperactive NSCLC in advanced‐stage disease.

## Materials and methods

### Ethics approval and patient consent

The study was approved by the Regional Medical Research Ethics Committee (297/2015) and by the Kuopio University Hospital Ethics Committee (315/2015) and was conducted in accordance with ethical principles outlined in the Declaration of Helsinki [18]. Informed consent was obtained from all study participants.

### Patient enrollment, sample processing, and sequencing

Patients with newly diagnosed inoperable lung cancer were prospectively enrolled at Kuopio University Hospital (2016–2023). Cohort details are shown in the supplementary material, Figure S1. Whole blood was collected from the peripheral arm vein into 10 ml EDTA‐vacutainer tubes at three time points: prior to therapy, at three‐month follow‐up, and after disease progression. Whole blood was centrifuged at 535×*g* (Heraeus Megafuge 1.0 centrifuge, Thermo Fisher Scientific, Waltham, MA, USA) for 20 min within 2 h from collection and stored at −80°C until further processing. Circulating cell‐free DNA was extracted from 5 ml of plasma using the Zymo Research cfDNA Serum & Plasma Kit (catalogue No: D4076, Zymo Research, Irvine, CA, USA) and quantified with Agilent 2100 BioAnalyzer (Agilent, Santa Clara, CA, USA) in conjunction with the Agilent High Sensitivity DNA kit according to the manufacturer's instructions. Libraries were prepared with the Agilent SureSelect XT kit custom gene panel either manually or using the automated Agilent Magnis NGS Prep System once available using 10 ng of input DNA in both instances. The resulting libraries were sequenced using the Illumina NextSeq 500 (Illumina, San Diego, CA, USA) with the 300‐cycle Mid Output Kit v2 or v2.5, as available.

### Processing of next generation sequencing (NGS) data

FASTQ files were processed with a custom pipeline adapted from Broad Institute's GATK Best Practices workflow for somatic short variant discovery (SNVs + Indels – https://gatk.broadinstitute.org/hc/en-us), implemented with Snakemake, using GATK v4.2.6.1, Picard v 2.27.1, (https://github.com/broadinstitute/gatk/releases, 13 August 2022) and BWA‐MEM v.2.2.1 (https://anaconda.org/channels/bioconda/packages/bwa-mem2/overview, 8 August 2022). In brief, quality control was performed on lane‐merged paired‐end FASTQ files using fastp with standard settings, with the ‐Q parameter applied to omit initial quality filtration. The resulting files were aligned to the hg38 reference genome using BWA‐MEM2. Aligned BAM files were restricted to the gene panel regions with SAMtools (https://anaconda.org/channels/bioconda/packages/samtools/overview, 8 August 2022). The unaligned reads were transferred to BAM format and merged with the aligned data using Picard FastqToSam and MergeBamAlignment, respectively. Data were sorted using SAMtools, and PCR and optical duplicates were tagged using Picard MarkDuplicates. Quality scores were recalibrated with GATK BaseRecalibrator and ApplyBQSR, and somatic mutations were called using Mutect2, controlling germline variants with a panel of normals and a germline resource from the standard GATK hg38 files. Read orientation bias was assessed with GATK LearnReadOrientationModel, and initial variant calls were filtered with GATK FilterMutectCalls using the following parameters: ‐distance‐on‐haplotype 0 ‐max‐events‐in‐region 4 ‐f‐score‐beta 2 ‐threshold‐strategy OPTIMAL_F_SCORE ‐min‐allele‐fraction 0.01 ‐min‐reads‐per‐strand 2. To harmonize genomic coordinates with external annotation tools, genome liftover to hg19 was conducted using Picard LiftoverVCF. Variants were annotated using GATK Funcotator, FATHMM‐MKL (https://github.com/HAShihab/fathmm-MKL, 13 August 2022) and OncoKB MafAnnotator (https://github.com/oncokb/oncokb-annotator, 9 February 2022).

To filter out PCR artifacts and likely germline calls, we first removed variants present in our platform‐specific panel of normal samples. Next, in addition to standard Mutect2 filtering, we applied hard filters for variants of unknown significance: ≥ 5 Mutect2 detected unique reads and less than 0.45 variant allele frequency (VAF). Less stringent evidence‐based filtering was applied for oncogenic and likely oncogenic variants, defined as having at least two supporting unique reads, and at least one of the following features: FATHMM‐score ≥ 0.99 in coding or non‐coding regions, categorically oncogenic, likely oncogenic or harboring a drug response in ClinVar (https://www.ncbi.nlm.nih.gov/clinvar/, 13 August 2022). Variants present in GnomAD and ExAC (https://gnomad.broadinstitute.org/, 5 March 2022) at > 1:100,000 frequency were removed to exclude remaining putative germline calls. Tumor mutation burden (TMB) was calculated from the resulting MAF file. Mutations in *NFE2L2* and *KEAP1* were reported at this stage. Co‐occurrence and mutual exclusivity for mutations were calculated for only the pathogenic and likely pathogenic variants, defined as before. To further dissect oncogenic *NFE2L2*, *KEAP1*, and *CUL3* mutations, additional *in silico* analysis was performed with The Cancer Genome Atlas (TCGA) data (see publicly available datasets section in Materials and methods).

### Molecular dynamics simulations and trajectory analysis

KEAP1 structures 8XGK and 9ETY were retrieved from the RCSB PDB (highest resolution with fully resolved T609 and M147/F190 residues, respectively). Point mutations p.T609K, p.M147V, and p.F190S were introduced in PyMOL (v. 2.5.0, Schrödinger LLC, New York, NY, USA). For 9ETY, the crystallographic KEAP1‐Nrf2 inhibitor was removed prior to system preparation.

All receptors were prepared using the Protein Preparation Wizard (distributed with Schrödinger Software Suite, v. 2024.3, Schrödinger LLC) with default settings, and termini were capped with methyl groups to avoid artifactual charged interactions. Molecular dynamics (MD) systems were set up with the Schrödinger Software Suite in an orthorhombic water box extending 15 Å from any protein atom, using the simple point charge water model. Systems were neutralized with Na^+^ or Cl^−^ ions and brought to physiological salt concentration (150 mm NaCl). Five independent replicas were simulated with Desmond for 2 μs each at 300 K, using the OPLS4 force field [19, 20].

MD trajectories of mutant and wild‐type receptors from 8XGK and 9ETY were compared with MDAnalysis (v. 2.7.0) [21]. Geometric similarity was quantified using directed Hausdorff distances, and the resulting distance matrices were clustered with Ward's hierarchical clustering [22]. Gaussian elastic network analysis was used to compare the conformational landscapes of wild‐type KEAP1 and mutant structures [23]. For geometric similarity and elastic network analysis, only α carbons were used with default settings. Elastic network eigenvalues from five replicates were sampled using non‐parametric bootstrapping (1000 iterations with replacement) for both mutant and wild‐type trajectories, and the resulting bootstrapped eigenvalue density functions were compared visually. Differences in average root mean square fluctuation (RMSF) values for wild‐type and mutant α carbons were further evaluated using Student's *t*‐test (equal variances, *α* = 0.05) with Bonferroni correction.

As 8XGK contained a competitive KEAP1‐Nrf2 inhibitor, we hypothesized that increased ligand instability upon introduction of an oncogenic point mutation may hint at mutation‐induced instability of the KEAP1‐Nrf2 interaction. Therefore, ligand root mean square deviation (RMSD) between wild‐type and mutant receptors was also compared and evaluated with Student's *t*‐test (equal variances, *α* = 0.05).

### Site directed mutagenesis and luciferase reporter assay

Point mutations (p.M147V, p.F190S, p.G480W, p.T609K) in human Keap1 were generated using the Phusion Site‐Directed Mutagenesis Kit (catalogue No: F541, Thermo Fisher Scientific). To introduce each mutation, the p3xFLAG plasmid containing the human *KEAP1* gene was amplified by PCR with primers carrying the specific mutations: p.M147V (Forward 5′‐ ggcctccatctccgtgggcgagaagtg‐3′, Reverse 5′‐cacttctcgcccacggagatggaggcc‐3′), p.F190S (Forward 5′‐catcggcatcgccaacagcgctgagcagattggc‐3′, Reverse 5′‐gccaatctgctcagcgctgttggcgatgccgatg‐3′), p.G480W (Forward 5′‐ggggggctttgactggacaaaccgcct‐3′, Reverse 5′‐aggcggtttgtccagtcaaagcccccc‐3′), p.T609K (Forward 5′‐ggcgtggctgtcaagatggagccctgcc‐3′, Reverse 5′‐ggcagggctccatcttgacagccacgcc‐3′) using Phusion Hot Start II DNA Polymerase.

The PCR products were digested with FastDigest DpnI, phosphorylated at the 5′ ends using T4 Polynucleotide Kinase (Thermo Fisher Scientific, catalogue No: EK0031), and then circularized through a ligation reaction. After ligation, the PCR products were transformed into DH5α competent cells via heat shock at 42°C for 40 s.

Single colonies were inoculated in lysogeny broth supplemented with ampicillin (100 μg/ml) for plasmid isolation using the NucleoSpin Plasmid EasyPure kit (catalogue No: 740727.50, MACHEREY‐NAGEL, Düren, Germany). Mutagenesis was confirmed by Sanger sequencing (Eurofins Genomics Europe Pharma and Diagnostics Products & Services Sanger/PCR GmbH, Cologne, Germany) using CMV‐Forward (5′‐CGCAAATGGGCGGTAGGCGTG‐3′) and hGH‐PA‐Reverse (5′‐CCAGCTTGGTTCCCAATAGA‐3′) primers.

HEK293 cells (3 × 10^5^) were seeded in 24‐well plates and co‐transfected with plasmids (pRL‐TK 30 ng, pGL3‐Promoter (2×) 180 ng or pGL3‐2 × GCLM‐ARE‐luc 180 ng, pCI‐HA‐Nrf2 100 ng, p3xFLAG hKeap1 CMV or p3xFLAG hKeap1 WT or p3xFLAG hKeap1 mutants (M147V, F190S, G480W, T609K) 200 ng) using Lipofectamine 3000 Transfection Reagent (Thermo Fisher Scientific, catalogue No: L3000008). After 16–24 h of transfection, a dual‐reporter assay was performed using the Dual‐Luciferase Reporter Assay System (catalogue No: E1910, Promega, Madison, WI, USA) following the manufacturer's protocol. Firefly luciferase activity was normalized to Renilla luciferase and expressed as Firefly/Renilla ratio. Statistical analysis was determined by one‐way ANOVA with multiple comparison using Graphpad Prism 10 (https://www.graphpad.com/updates, GraphPad Software LLC, Boston, MA, USA) (*p < 0.05; **p < 0.01; n.s, not significant).

### Immunohistochemistry

IHC for AKR1B10 and Ki67 were performed on the Leica Bond III staining system (Leica Biosystems, Nussloch, Germany) using BOND Polymer Refine Detection kit (catalogue No: DS9800, Leica Biosystems) and BOND Epitope Retrieval Solution 2 (Leica Biosystems, catalogue No: AR9640) with 20 min incubation. AKR1B10 Antibody (Clone 1A6, catalogue No: H00057016‐M01, Abnova, Taipei, Taiwan) and Ki67 (clone SP6, catalogue No: MA5‐14520, Invitrogen, Waltham, MA, USA) were used as 1:100 dilution in BOND primary antibody diluent and staining was conducted following the manufacturer's standard protocol for Bond III IHC.

AKR1B10 was categorically assessed from the resulting slides in a blinded manner (by LM). The classes were defined as strong expression (3), moderate or variable expression (2), low expression (1), and negative (0). Ki67 was assessed by annotating proliferative epithelial hotspot regions and running QuPath's *positive cell detection* algorithm with default parameters and computing the proportion of positive cells from all tumor cells.

Multiplex immunohistochemistry (mIHC) for markers PD‐1 (SP‐269), CD45 (X17/99), CD56 (MRQ‐42), CD8 (4B11), GCLC (HPA035359), CD3 (LN10), AKR1C1 (AB192785), and pan‐cytokeratin (BS5) was run using the same staining system. DAB‐chromogen was switched to 3‐amino‐9‐ethylcarbazole (catalogue No: ab103742, AEC Single/Plus, Abcam, Cambridge, UK), and the protocol and data analysis were performed as previously described [24]. In brief, after each staining cycle, the tissue slides were mounted with VectaMount AQ aqueous mounting medium (H‐5501‐60) and scanned. Ethanol was used to destain the slides, and heat‐mediated antibody stripping was applied before subsequent staining cycles. All IHC‐stained samples were digitized with Hamamatsu NanoZoomer XR (Hamamatsu Photonics, Shizuoka, Japan) with 20× magnification.

### Computational image analysis

All image analyses were performed with QuPath v.0.4.3 [25]. The tissues were divided into 40 representative patches of 500 × 500 pixels (0.45 μm/pixel), and 5–10 representative areas were annotated for each patch to generate training data. For the segmentation of tissue regions, tumor was defined as pan‐cytokeratin positive, stroma as pan‐cytokeratin negative, whereas necrosis, artefacts (such as tissue folds) or empty areas were set to ignore. For cell identification, the cells were classified as tumor (pan‐cytokeratin+), leukocyte (CD45+), and other (intact nuclei, negative for all antibodies).

Tumor, stroma, and artefactual or necrotic areas were segmented with QuPath's random trees pixel using resolution of 0.9 μm/pixel, scales 1 and 4 and the following features: Gaussian, Laplacian of gaussian, weighted deviation, and gradient magnitude (everything else set to default). Cell classification was conducted with QuPath's random trees object classifier. Cell detection was conducted with sigma of 1.2 μm. Representative patches (selected in a blinded manner by LM) were defined for each sample, and QuPath's classifiers were run for the respective regions. For the resulting data, the leukocytes were further classified by marker expression. The populations were defined as T cells (CD3+), CD8+ T cells (CD3+; CD8+), NK cells (CD56+), and other leukocytes (defined as leukocytes by QuPath but negative for the previously listed markers).

### Visualization

Visualization was implemented with QuPath (v.0.4.3), ggplot2 (v.3.5.0), ComplexHeatmap (v.2.18.0), maftools (v.2.18.0), and survminer (v.0.5.0.999) R‐packages. All analyses were conducted with RStudio version 2022.7.2 (https://posit.co/downloads/, 25 September 2022) and R version 4.2.1 (https://cran.r-project.org/bin/windows/base/, 25 September 2022).

### Publicly available datasets

Clinical and genomic data from Memorial Sloan Kettering Cancer Center (MSK) cohorts were downloaded from https://www.cbioportal.org/ [26, 27, 28]. TCGA mRNA expression and mutation data were obtained from the Pan‐Cancer Atlas (https://gdc.cancer.gov/about-data/publications/pancanatlas) [29]. For the MSK‐CHORD cohort, only NSCLC cases were used, and for the metastatic NSCLC, other than ctDNA mutations were filtered from the maf‐file. From the TCGA mutation data, silent variants were excluded from the analysis. Shannon TCR entropy and richness were obtained from Thorsson *et al* (2018) [30].

NRF2 scores for TCGA samples were computed as previously described [31]. NRF2 score was assessed for codons present in the prospective cohort, and variants having a higher mean score than the NRF2 hyperactivity threshold were defined as activating. For NRF2‐pathway mutations, all detected non‐silent mutations in *NFE2L2*, *KEAP1*, and *CUL3* were classified as mutated.

Deconvolution for cellular content was conducted for TCGA data with immunedeconv R‐package using the QuanTIseq‐method. Samples were clustered with ComplexHeatmap based on M1/M2 macrophages, neutrophils, regulatory, B, T, NK, and dendritic cells.

Differential expression was performed with limma (v.3.58.1) following the user manual (Chapter 15) [32]. Gene‐set enrichment analysis was performed with fgsea (v.1.28.0) R‐package with the Hallmark gene set collection from the Molecular Signatures Database [33, 34].

### Statistical analyses

For clinical associations with binary outcome, univariable and multivariable logit models were used for the prospective cohort and public TCGA data, respectively. Co‐occurrence and mutual exclusivity of mutations were assessed with Fisher's exact test, and differences between individual continuous variables against a binary variable were evaluated with two‐sided Mann–Whitney U tests. The significance of clinical outcomes was assessed using log‐rank tests. Survival analyses were conducted using the survival R‐package. Receiver operating characteristic (ROC) analyses and metric optimization using the maximum Youden's J statistic were performed using the PRROC (https://CRAN.R-project.org/package=PRROC, 25 September 2022) and cutpointr (https://CRAN.R-project.org/package=cutpointr, 25 September 2022) R‐packages.

## Results

### High plasma cfDNA levels persist until disease progression in patients with inoperable NSCLC

The initial cohort comprised 80 inoperable NSCLC cases, each with at least one blood sample. Samples were collected at diagnosis, at 3‐month follow‐up, and at disease progression (median follow‐up time: 40.4 months).

Mean plasma cfDNA concentration was 27.6 ng/ml (range 1.8–685.0; supplementary material, Figure S1A). No evaluated clinical variable (stage, smoking status, age, or histology) explained this variability, although patients with stage IV disease had the highest cfDNA levels, consistent with prior reports [35, 36].

cfDNA concentrations at follow‐up and progression correlated positively with baseline levels (*p* < 0.0001 and *p* = 0.004, respectively; supplementary material, Figure S1B,C). No significant changes were observed across timepoints upon pairwise testing, suggesting that drivers of elevated cfDNA persist throughout disease course in patients with inoperable NSCLC.

### Mutations in *NFE2L2* , *KEAP1* , and *CUL3* are associated with male sex, prior smoking history, and negative *EGFR* status

Targeted NGS of 75 lung cancer‐associated genes were successfully performed in 73 cases at baseline (genomic regions are listed in the supplementary material, Table S2; average deduplicated read depth in supplementary material, Figure S1D; cohort details in supplementary material, Table S1). Of these patients, 14 (19%) exhibited coding mutations with ≥ 1% VAF (excluding silent variants) in *KEAP1*, *NFE2L2*, or *CUL3*. To identify mutations that potentially activate the NRF2 pathway, we first excluded mutations likely leading to LOF in *NFE2L2* (a single case with a frameshift mutation in the Neh2 domain).

For the remaining variants, we evaluated NRF2 activity using the NRF2 score [31] for corresponding codons present in the TCGA data. All missense variants with matching codon alterations in TCGA data exceeded the previously defined threshold for NRF2 hyperactivity, suggesting likely functional variants (supplementary material, Figure S1E). Additionally, truncating and frameshift mutations in *KEAP1* and *CUL3* were considered likely NRF2‐activating events. Two *KEAP1* variants, p.T609K and p.F190S, lacked matching codon data in TCGA, thus precluding NRF2 activity evaluation. All the variants except the NRF2 LOF mutation were classified as either bona fide (hotspot) or putative NRF2‐activating mutations, totaling 13 cases and representing 18% of the cohort. Details of mutations and histological information are provided in Table 1.

**Table 1:** NRF2 pathway mutations in the study cohort. Predicted effects on NRF2 activity were classified as activating when the respective codon harbored a median NRF2 score above the hyperactivity threshold in TCGA, or as likely activating when a loss‐of‐function event was observed in *KEAP1* or *CUL3*.

Gene	cDNA change	Protein change	VAF %	Predicted relation to NRF2 activity	Histology	Differentiation
*NFE2L2*	c.(181‐183)Gat>Cat	p.D77H	12.1	Activating	LUSC	‐
*NFE2L2*	c.52_53ins	34 in‐frame ins	2.1	Activating	LUSC	Keratinizing
*KEAP1*	c.832C>G/c.425C>T	p.P278A	5.5	Activating / unknown	LUSC	Non‐keratinizing
*KEAP1*	c.1826_1827CC>AA	p.T609K	10.5	Unknown	NSCLC, NOS	‐
*KEAP1*	c.967_968AA>GC	p.K323A	3.0	Activating	LUAD	‐
*KEAP1*	c.967_968AA>GC	p.F190S	2.9	Unknown	LUSC	Non‐keratinizing
*KEAP1*	c.1438G>T	p.G480W	7.6	Activating	LUSC	Non‐keratinizing
*CUL3*	c.2251A>T	p.R751*	1.9	Likely activating	NSCLC, NOS	‐
*NFE2L2*	c.22 T>C	p.W24R	7.7	Activating	LUAD	‐
*NFE2L2*	c.181G>C	p.D77H	5.4	Activating	LUSC	Non‐keratinizing
*KEAP1*	c.439A>G	p.M147V		Unknown	LUAD	‐
*KEAP1*	c.646A>T	p.K216*	2.1	Likely activating	NSCLC, NOS	‐
*KEAP1*	c.1237delC	p.R413fs	30.0	Likely activating	LUSC	Not available
*NFE2L2*	c.41_44del/c.33_34ins	p.I28fs / p.L30fs	4.1/4.0	Likely LOF	NSCLC, NOS	‐

cDNA, complementary DNA; LOF, loss of function; LUAD, lung adenocarcinoma; LUSC, lung squamous cell carcinoma; NOS, not otherwise specified; NRF2, nuclear factor erythroid 2‐related factor 2; NSCLC, non‐small cell lung cancer; TCGA, The Cancer Genome Atlas; VAF, variant allele frequency.

Next, we analyzed the clinical characteristics of patients with NRF2 pathway mutations. All mutation‐positive cases were male, presented with advanced‐stage disease, and had a history of smoking. Data from TCGA confirmed that male sex and smoking history are associated with a higher risk of harboring NRF2 pathway mutations in lung adenocarcinoma (LUAD) (OR = 1.96, *p* < 0.001 and OR = 3.07, *p* < 0.05, respectively), but this was not observed in lung squamous cell carcinoma (LUSC). NRF2 pathway mutations did not overlap with diagnostic EGFR driver mutations in our cohort. TCGA analysis showed mutual exclusivity between EGFR and NRF2 pathway mutations (*p <* 0.0001). Diagnostic clinicopathological data and their statistical associations are shown in Table 2.

**Table 2:** Clinical characteristics and NRF2 pathway mutations in the study cohort.

Characteristic	*NFE2L2*/*KEAP1* mutation*	Association (Fisher's exact test or logit^†^)	TCGA LUAD association^†^	TCGA LUSC association^†^
All cases	13/73 (16%)			
Sex				
Male	13/53 (25%)	*p* = 0.01	OR_male_ = 1.96; *p <* 0.001	NS
Female	0/20 (0%)			
Age (years)				
< 65	3/18 (16%)			
≥ 65	10/55 (18%)	NS	NS	NS
Histology				
Squamous cell cancer	6/29 (21%)			
Adenocarcinoma	4/32 (13%)	NS	‐	‐
NSCLC, other	3/12 (25%)			
AJCC stage				
I–II	0/5 (0%)			
III–IV	13/68 (19%)	NS	NS	NS
Smoking status				
Never smoker	0/11			
Ex‐smoker	7/32 (21%)	*p* = 0.19^‡^	OR = 3.07; *p* < 0.05	NS
Current smoker	6/29 (21%)			
PD‐L1				
> 50%	2/13 (3.5%)			
1–49%	7/25 (28%)	NS	‐	‐
< 1%	1/19 (5%)			
ND	3/16 (19%)			
Immunotherapy				
Yes	2/14 (14%)			
No	11/58 (18%)	NS	‐	‐
EGFR (Idylla)				
Yes	0/7 (0%)			
No	6/37 (16%)	NS	OR_EGFR_ = 0; *p* < 0.0001	OR_EGFR_ = 0.18; *p* = 0.09

AJCC, American Joint Committee on Cancer; ctDNA, circulating tumor DNA; LUAD, lung adenocarcinoma; LUSC, lung squamous cell carcinoma; ND, not determined; NRF2, nuclear factor erythroid 2‐related factor 2; NSCLC, non‐small cell lung cancer; PD‐L1, programmed death‐ligand 1; TCGA, The Cancer Genome Atlas.

* Detected in ctDNA.

^†^ Fisher's and logit for categorical and continuous or ordinal independent variable, respectively.

^‡^ Measured as two‐class endpoint (never versus ever smoker).

### Circulating NRF2‐activating mutations are highly indicative of NRF2 activation in the primary tumor and associate with aggressive course of disease

**Figure 1:**
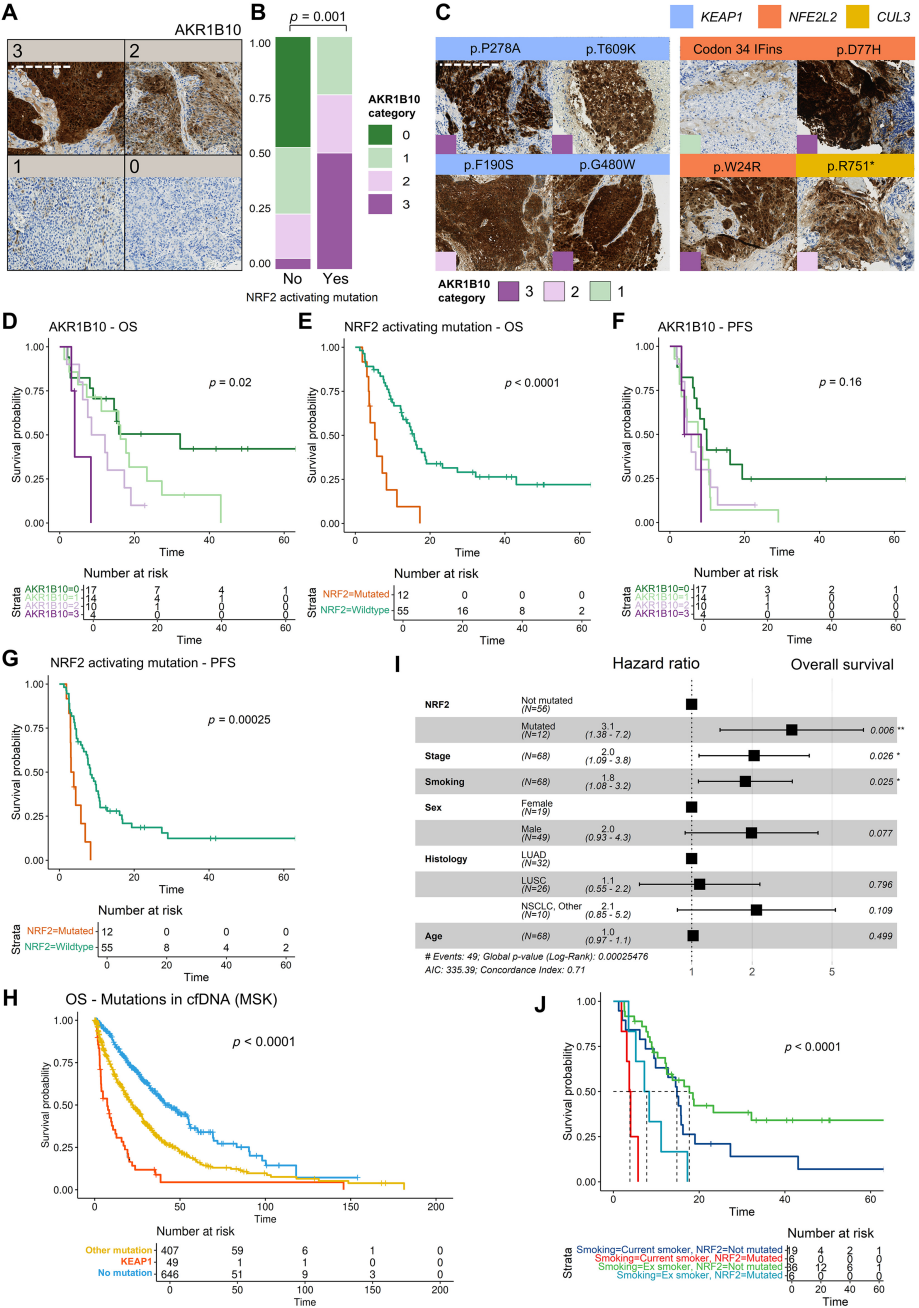
Clinical consequences of nuclear factor erythroid 2‐related factor 2 (NRF2) activation. (A) Representative images of AKR1B10 protein expression categories. Scale bar, 250 μm. (B) Proportions of AKR1B10 protein expression categories between ctDNA NRF2 pathway mutated and wild‐type cases. (C) Representative image patches of AKR1B10 protein expression in NRF2 pathway mutated cases. Scale bar, 250 μm. (D) Overall survival (OS) across AKR1B10 categories. (E) OS between NRF2 pathway mutated and non‐mutated cases. (F) Progression‐free survival (PFS) across AKR1B10 categories. (G) PFS between NRF2 pathway mutated and non‐mutated cases. (H) OS for circulating *KEAP1* mutations (orange), any other mutations (yellow), and no mutations (blue). (I) Multivariable Cox regression analysis for OS with NRF2 pathway mutations, clinical stage, sex, histology, and age as predictors. (J) OS between current smokers and ex‐smokers in NRF2‐mutated and wild‐type cases. cfDNA, cell‐free DNA.

To determine the functional impact of mutations detected in ctDNA, we evaluated AKR1B10 (a canonical NRF2 target) protein expression by IHC in core needle biopsies. Staining intensity in cancer cells was evaluated blindly and categorized visually between classes 0 and 3 as illustrated in Figure 1A. AKR1B10 expression associated strongly with NRF2 pathway mutations (*p* = 0.001, Fisher's exact test; Figure 1B). We additionally measured the mean intensity per cell of AKR1B10 at representative regions, where digital intensity compared with human assessment showed similar discriminatory value (area under the curve (AUC) values of 0.87 and 0.86, respectively; supplementary material, Figure S1F–H). By maximizing Youden's metric, optimal cut‐off point for AKR1B10 was ≥ 2. Using this rationale to categorize NRF2 overexpression, AKR1B10 demonstrated 78% accuracy (with 78% sensitivity and 78% specificity) relative to NRF2 pathway variants in ctDNA. Notably, the novel variants p.T609K and p.F190S also exhibited strong AKR1B10 staining. Furthermore, a LOF mutation in *CUL3* was similarly associated with AKR1B10 positivity (Figure 1C). Expectedly, the only case with strong AKR1B10 staining but no detectable mutations had a localized tumor, likely indicative of ctDNA concentrations below our VAF detection threshold of 1%.

Beyond the associations with target protein activation, we explored the three novel *KEAP1* mutations, p.T609K, p.F190S, and p.M147V with computational and *in vitro* methods. Our molecular dynamics simulations showed a marked shift in the dynamic trajectories for p.T609K (supplementary material, Figure S2A), but not for p.F190S (data not shown), in four separate simulation experiments. Based on the differences in per‐residue RMSFs in the p.T609K structures, a significant change was associated with residue 367 and the loop with residues 397–401 (supplementary material, Figure S2B). Upon further assessing the mutations' impact on molecular dynamics with Gaussian elastic network analysis, both mutations resulted in shifts from the wild‐type conformational landscape (supplementary material, Figure S2C,D). While the changes for p.T609K may arise from the given residues, p.F190S shows a strong tendency towards a single conformation in contrast to the bimodal distribution of wild‐type KEAP1, deviating also from the prominent peak associated with the crystallographic structure (supplementary material, Figure S2D,E). Since the given structure exists as a homodimer, this conformational shift may affect the dimerization of KEAP1. In our simulations, the p.T609K mutation did not affect the RMSD of the crystallographic competitive KEAP1‐NRF2 inhibitor, suggesting that NRF2 binding is not affected by this mutation, and changes in NRF2 activity may rather be explained by changes in other protein–protein interactions arising from conformational shifts (including deterred KEAP1 dimerization for p.F190S). Mutation p.M147V did not show structural/dynamic shifts in the applied analyses and was therefore considered a putative passenger event (supplementary material, Figure S2F,G). Finally, we aimed to further validate these observations *in vitro* using site‐directed mutagenesis, to both benchmark our *in silico* approach and highlight the value of AKR1B10 in verifying functional NRF2‐pathway variants. The mutagenesis experiments showed that KEAP1 mutations p.T609K and p.F190S, but not p.M147V, disrupted the ability of KEAP1 to inhibit NRF2 activity (supplementary material, Figure S2H), aligning with our *in silico* analysis. Based on these data, we excluded p.M147V from the subsequent analyses.

After confirming NRF2 activation, we examined clinical outcomes of patients with late‐stage disease and NRF2 pathway mutations and/or immunopositivity. Both AKR1B10 expression in tumor and NRF2 activating mutations in ctDNA were associated with inferior overall survival (OS) (Figure 1D,E). Additionally, NRF2‐activating mutations, but not AKR1B10 expression, were predictive of earlier disease progression (Figure 1F,G). Median overall survival (mOS) was 5.2 versus 15.7 months (*p* < 0.0001), and median progression‐free survival (mPFS) was 3.5 versus 8.3 months (*p* = 0.0003) for patients with circulating NRF2‐activating mutations compared with those without, respectively. As the prognostic effect of the presence of any versus no ctDNA alterations has been established [28], we further confirmed that NRF2 pathway mutations in ctDNA are associated with inferior survival relative to both any and no ctDNA alterations: in the MSK metastatic NSCLC cohort, mOS was 7.4 months for NRF2 pathway mutations, 22.1 months for any other mutation, and 41.45 months for mutation‐free cases (*p* < 0.0001; Figure 1H). When adjusted for age, stage, smoking, sex, histology, and age, NRF2 pathway mutation status in ctDNA was an independent predictor of OS (HR = 3.1; *p* = 0.009) but was not independently associated with disease progression (Figure 1I; supplementary material, Figure S3A). As our cohort included only one case with NRF2 pathway mutation that was treated with immune‐checkpoint blockade (ICB), we could not assess the independent effect of the presented mutations and immunotherapy responses. However, our study included five cases with AKR1B10 immunopositivity and ICB treatment; thus, we controlled for treatment in assessing OS with multivariable cox regression. Based on our analysis, high AKR1B10 expression was independently associated with poor survival, while ICB was an independent predictor of favorable outcome (supplementary material, Figure S3B,C; see Limitations section for further remarks).

Collectively, these findings indicate that NRF2 overexpression is independently associated with aggressive disease outcomes in NSCLC. Additionally, smoking correlates with NRF2 pathway alterations and poor prognosis, suggesting its potential role in aggressive disease progression via NRF2 activation. However, as smoking remains an independent prognostic factor, cessation should still improve survival in NRF2‐driven NSCLC (Figure 1J). Importantly, these results highlight IHC of AKR1B10 as a robust quantifiable biomarker for assessing functionality of ambiguous findings and suggest that in addition to the hotspots and truncating *KEAP1* aberrations, the two rare mutations (*KEAP1* p.T609K and p.F190S) dysregulate the NRF2 pathway and should be considered pathogenic high‐risk variants.

### Circulating NRF2 mutations co‐occur with SMARCA4 alterations and high blood‐based tumor mutational burden, cumulatively worsening prognosis

Next, we aimed to characterize the mutational landscape of pathogenic or likely pathogenic coding mutations among significant tumor suppressor genes and oncogenes in our cohort, employing evidence‐based and normal panel‐based filtering methods (see Materials and methods). Before filtering, we calculated the blood‐based tumor mutational burden (bTMB) from all detected mutations [37]. We benchmarked bTMB as a surrogate for TMB by evaluating its association with (a) total mutation burden in TCGA within the corresponding genomic regions, and (b) smoking history within our cohort [38]. In lung cancer, TMB among the genes represented in the panel robustly predicted total exome TMB (*R* = 0.89 *p* < 0.0001; supplementary material, Figure S4A). In addition, median bTMB increased from never to ever and current smokers in lung adenocarcinoma within our cohort (*p* = 0.038 and 0.037, respectively; supplementary material, Figure S4B).

**Figure 2:**
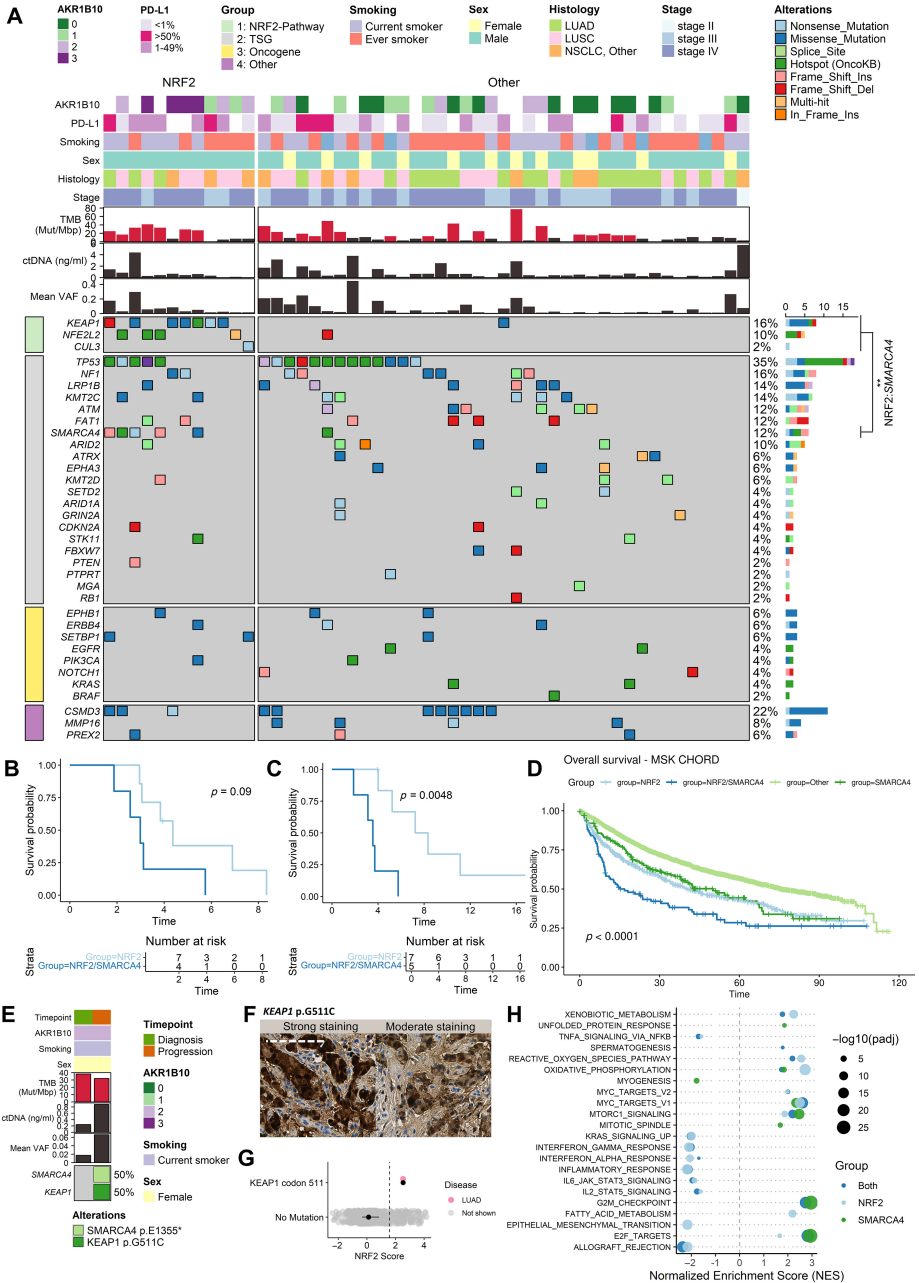
Molecular features and prognostic implications of nuclear factor erythroid 2‐related factor 2 (NRF2) pathway and *SMARCA4*‐mutated cases. (A) The mutational landscape of diagnosis timepoint samples. Mutation frequency is shown on the right‐hand panel, and clinicopathological variables are shown in the top panel. Co‐event analysis with Fisher's exact test indicates that *SMARCA4* mutations co‐occur with NRF2 pathway mutations (*p* < 0.01). (B) Progression‐free survival between *SMARCA4*‐mutated and non‐mutated cases within the NRF2 pathway mutated population. (C) Overall survival between *SMARCA4*‐mutated and non‐mutated cases within the NRF2 pathway mutated population. (D) Overall survival across *SMARCA4* and NRF2 pathway co‐mutated, NRF2 pathway, or *SMARCA4*‐mutated cases. (E) Clinicopathological and characteristics of a case with *KEAP1*/*SMARCA4* mutations emerging at disease progression. The case with *KEAP1* and *SMARCA4* mutations at progression is outlined in red. (F) AKR1B10 expression of a case with circulating NRF2 pathway mutation and *SMARCA4* mutation emerging at progression timepoint. Scale bar, 125 μm. (G) NRF2 activity score in *KEAP1* p.G511C in TCGA. (H) Enriched hallmark pathways across *SMARCA4* and NRF2 pathway co‐mutated, NRF2 pathway, or *SMARCA4*‐mutated cases in TCGA. ctDNA, circulating tumor DNA; LUAD, lung adenocarcinoma; LUSC, lung squamous cell carcinoma; NSCLC, non‐small cell lung cancer; PD‐L1, programmed death‐ligand 1; TCGA, The Cancer Genome Atlas; TMB, tumor mutational burden; TSG, tumor suppressor gene; VAF, variant allele frequency.

After filtering out silent non‐pathogenic variants, we determined ctDNA concentration as the product of cfDNA concentration and mean VAF for each case (VAF distribution for pathogenic variants is shown in supplementary material, Figure S4C). The most frequently mutated genes detected in ctDNA aligned closely with known mutations in NSCLC, identifying *TP53*, *CSMD3*, *KEAP1*, *NF1*, and *LRP1B* as the top five mutated genes (Figure 2C). NRF2 pathway mutations co‐occurred significantly with *SMARCA4* mutations (*p* < 0.01; Figure 2A), a finding confirmed by TCGA data showing significant co‐occurrence in LUAD (supplementary material, Figure S4D). In addition, circulating NRF2 mutations correlated with higher bTMB (supplementary material, Figure S4E). TCGA analysis demonstrated that primary NRF2 pathway mutations, *SMARCA4* mutations, and their co‐occurrence correlated with high TMB in LUAD, whereas only co‐mutated cases exhibited higher mutation counts in LUSC (supplementary material, Figure S4F,G).

Co‐occurring *SMARCA4* and NRF2 pathway mutations were associated with significantly worse mOS compared with NRF2 pathway mutations alone (Figure 2B,C). This finding was supported by the MSK‐CHORD NSCLC cohort, where mutations in NRF2 pathway and *SMARCA4* independently predicted poorer outcomes, which were cumulatively worsened when both mutations co‐occurred (Figure 2D). Multivariable analysis in MSK‐CHORD confirmed that NRF2 pathway mutations, *SMARCA4* mutations, smoking status, and disease stage independently predicted inferior outcomes (supplementary material, Figure S4H). Multivariable logistic regression analysis revealed that smoking independently increased the likelihood of NRF2 pathway and *SMARCA4* mutations (*p* < 0.0001 for both), while sex did not.

At follow‐up, four of the 12 NRF2 mutation‐positive cases remained alive. Among these, one retained the circulating KEAP1 truncating mutation, and two were mutation free. No new NRF2 or SMARCA4 mutations emerged during follow‐up. Notably, surviving cases initially exhibiting NRF2 activation had moderate to low AKR1B10 expression by immunohistochemistry, whereas those with strong expression had decreased. Combined with AKR1B10 survival analysis (Figure 1B), these findings suggest that variance in NRF2 activity or target gene profiles within the mutationally activated hyperactive population have prognostic implications. At disease progression, a new *KEAP1*/*SMARCA4* co‐mutated (p.G511C and p.E1355*) case emerged displaying with similar clinical features as the prior NRF2 pathway mutated cases, in those currently smoking and having a poor outcome; deceased 31 days after obtaining the progression timepoint sample (Figure 2E). Given that the median survival time from progression to death was 174 days, this outcome aligned with the previously observed increased hazard in NRF2‐mutated cases (Figure 1G). AKR1B10 IHC showed strong to moderate variable staining, suggesting clonal NRF2 activation at diagnosis (Figure 2F). TCGA data confirmed that *KEAP1* codon 511 scored above the hyperactivity threshold (Figure 2G).

Our cohort included 17 cases available for monitoring tumor evolution, with mutations observed in matching diagnostic and follow‐up timepoints. Of these, seven cases harbored at least one recurring circulating oncogenic mutation at follow‐up. Recurring mutations at follow‐up predicted rapid disease progression (mPFS 3.5 versus 7.7; *p* = 0.0057) and trended toward worse OS (mOS 9.3 versus 17.2 months; *p =* 0.16), potentially reflecting treatment‐resistant dominant clones or higher disease burden (supplementary material, Figure S5A–C).

Given the significant overlap between *SMARCA4* and NRF2‐activating mutations, and their co‐occurrence deteriorates outcome, we explored their associations with biological signaling pathways in TCGA‐LUAD. *SMARCA4* mutations alone elevated NRF2‐activity, but not as robustly as NRF2 pathway mutations (supplementary material, Figure S5D). From cancer hallmarks, both pathways were associated with proliferation (MYC/MTOR/E2F), while only NRF2 was associated with decreased inflammatory pathways (IFNγ/STAT5/STAT3; Figure 2H). To further explore the effects of these aberrations on proliferation, we assessed Ki67 from the tumor tissue. Co‐mutated cases showed a trend towards higher proliferation index (51–75%; *p* = 0.12, supplementary material, Figure S5E), and AKR1B10 expression level trended towards increased Ki67 proliferation index (*p* = 0.06; supplementary material, Figure S5F). High Ki67 in advanced cases was associated with poor outcome independently of NRF2 (supplementary material, Figure S5G,H).

Collectively, these data suggest NRF2 activation as an early oncogenic event associated with SMARCA4 loss, each independently worsening disease outcomes and driving aggressive clinical course. Smoking independently increased the risk of both mutation types and negatively impacted survival. Transcriptomic profiling suggested that NRF2 activation and SMARCA4 loss jointly promote aggressive cancer cell proliferation and enhanced antioxidant and anti‐xenobiotic defenses in NSCLC.

### TMB affects cytotoxic T‐cell infiltration regardless of NRF2 pathway mutation status

**Figure 3:**
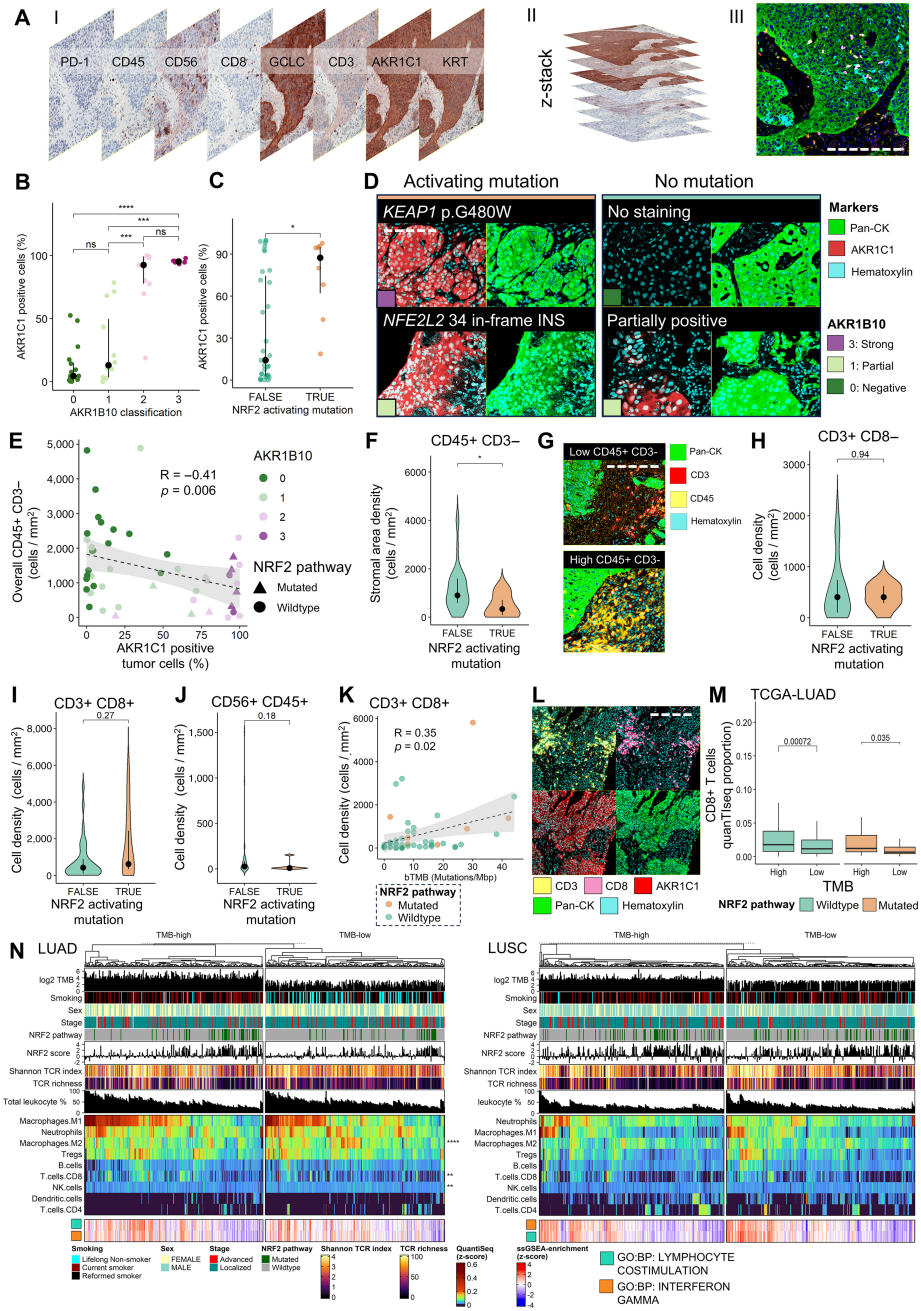
AKR1C1 protein expression and immunological features in nuclear factor erythroid 2‐related factor 2 (NRF2) hyperactive cases. (A) Overview of the multiplex immunohistochemistry (mIHC) workflow in tissue staining. Scale bar, 250 μm. (B) AKR1C1 protein expression in AKR1B10 expression categories. (C) AKR1C1 protein expression between NRF2 pathway mutated and non‐mutated cases. (D) Representative images of AKR1C1 protein expression in NRF2 pathway mutated and non‐mutated cases. Scale bar, 125 μm. (E) Scatter plot of AKR1C1 positive tumor cell percentage and non‐T/NK leukocyte density. (F) Non‐T/NK leukocyte density between NRF2 pathway mutated and non‐mutated cases. (G) Representative image patches of high‐density and low‐density cases. (H) Non‐cytotoxic T‐lymphocyte density between NRF2 pathway mutated and non‐mutated cases. (I) Cytotoxic T lymphocytes between NRF2 pathway mutated and non‐mutated cases. (J) NK cell density between NRF2 pathway mutated and non‐mutated cases. (K) Scatter plot of blood‐based tumor mutational burden (bTMB) and CD8+ T‐cell infiltration. (L) Case harboring high tumor mutation burden (TMB), NRF2 activation by somatic mutation of *KEAP1*, and intraepithelial and stromal cytotoxic T lymphocyte infiltration. Scale bar, 250 μm. (M) Association of TMB and deconvoluted CD8+ T‐cell proportion in The Cancer Genome Atlas (TCGA) lung adenocarcinoma (LUAD). (N) Heatmap of deconvoluted immune cells in TCGA LUAD and lung squamous cell carcinoma (LUSC). The top panel depicts TMB, smoking status, sex, stage, NRF2 pathway mutation status, and activity score, as well as T‐cell receptor Shannon index and richness. The middle panel depicts QuanTIseq‐deconvoluted cell proportions, and the bottom panel shows single sample Gene Set Enrichment Analysis (ssGSEA) scores for interferon gamma and lymphocyte costimulation Gene Ontology (GO)‐terms. GO:BP, Gene Ontology: biological pathway.

Given the link between NRF2 activation and immune evasion, we evaluated how NRF2 pathway mutations interact with immune cell infiltration, particularly in the context of TMB. We used a mIHC panel including CD45, CD3, CD8, CD56, AKR1C1, GCLC, and pan‐cytokeratin to assess NRF2‐tumor‐immune interactions in 47 cases (workflow, Figure 3A).

To validate the NRF2 markers included in our mIHC panel (AKR1C1 and GCLC), we compared their expression against AKR1B10 expression and NRF2 pathway mutations. AKR1C1 expression significantly correlated with both AKR1B10 and NRF2 pathway mutations, while GCLC did not (Figure 3B–D, and supplementary material, Figure S6A). The AUC for AKR1C1 mean cellular intensity in predicting circulating NRF2 mutations was 0.78 (supplementary material, Figure S6B), demonstrating a maximum accuracy of 0.66, with 100% sensitivity and 59% specificity. Notably, one *NFE2L2*‐mutated case with negative AKR1B10 staining showed strong AKR1C1 positivity, supporting the utility of multi‐marker assessment (Figure 3D, lower left). Another mutation‐negative case showed partial expression of both markers.

NRF2 hyperactivation was associated with reduced densities of non‐T/NK leukocytes (CD45+, CD3−, CD56−) in tumors (*R* = −0.41, *p* = 0.02; Figure 3E–G). In contrast to previous studies, NRF2 activation was not significantly associated with overall T‐cell (CD3+) or CD8+ T‐cell densities in our cohort (Figure 3H,I). Although not statistically significant, there was a trend towards lower NK cell density (*p* = 0.18, Figure 3J). Supporting these findings, TCGA data analyzed with QuanTIseq showed decreased NK cell fractions in NRF2 hyperactive NSCLC (supplementary material, Figure S6C,D). Additionally, consistent with previous reports, NRF2 pathway mutations in TCGA NSCLC correlated with lower CD8+ T‐cell infiltration (supplementary material, Figure S6E,F).

Given the discrepancy in CD8+ T‐cell density in our data compared with prior reports, and considering the positive association between TMB and CD8+ T‐cell infiltration, we investigated the independent contributions of bTMB and NRF2 activation to CD8+ T‐cell infiltration. Linear modeling indicated that bTMB was positively associated with CD8+ T‐cell infiltration independently of NRF2 activation (supplementary material, Figure S6G), showing a modest but significant correlation (*R* = 0.35, *p* = 0.02; Figure 3K,L).

To further explore this relationship, we evaluated the relationship between TMB, NRF2 pathway mutations, and cytotoxic immune responses in TCGA. Notably, CD8+ T‐cell proportions were elevated in TMB‐high cases in LUAD in both NRF2 pathway mutated and wild‐type groups (*p =* 0.035 for NRF2 activated and *p* = 0.0007 for NRF2 wild type; Figure 3M), but not in LUSC (supplementary material, Figure S6H). However, when examining the immunological milieu separately in TMB‐high and TMB‐low groups by hierarchical clustering of cellular content, NRF2 pathway mutated tumors fell into clusters with similar attributes: overall downregulation of proinflammatory signaling and costimulatory genes, as well as lower populations of myeloid and lymphoid cells, and less variation in T‐cell receptor repertoire (Figure 3N). In LUAD, TMB was associated with a tumor microenvironment (TME) more aligned with adaptive/antigen‐stimulated immune responses; increased cytotoxic T cells, as well as decreased M2‐type macrophages and NK cells (Figure 3N, left panel). TMB and smoking status did not alter the immunological landscape of LUSC, and NRF2 pathway mutated cases enriched towards the immune‐cold clusters in both groups (Figure 3N). Despite LUSC displaying a generally higher baseline TMB, LUAD showed greater variability in mutation burden (Figure 3N, top panel). Taken together, these data suggest that high neoantigen loads may partially offset the reduction in cytotoxic lymphocytes in NRF2 hyperactive lung cancer.

## Discussion

**Figure 4:**
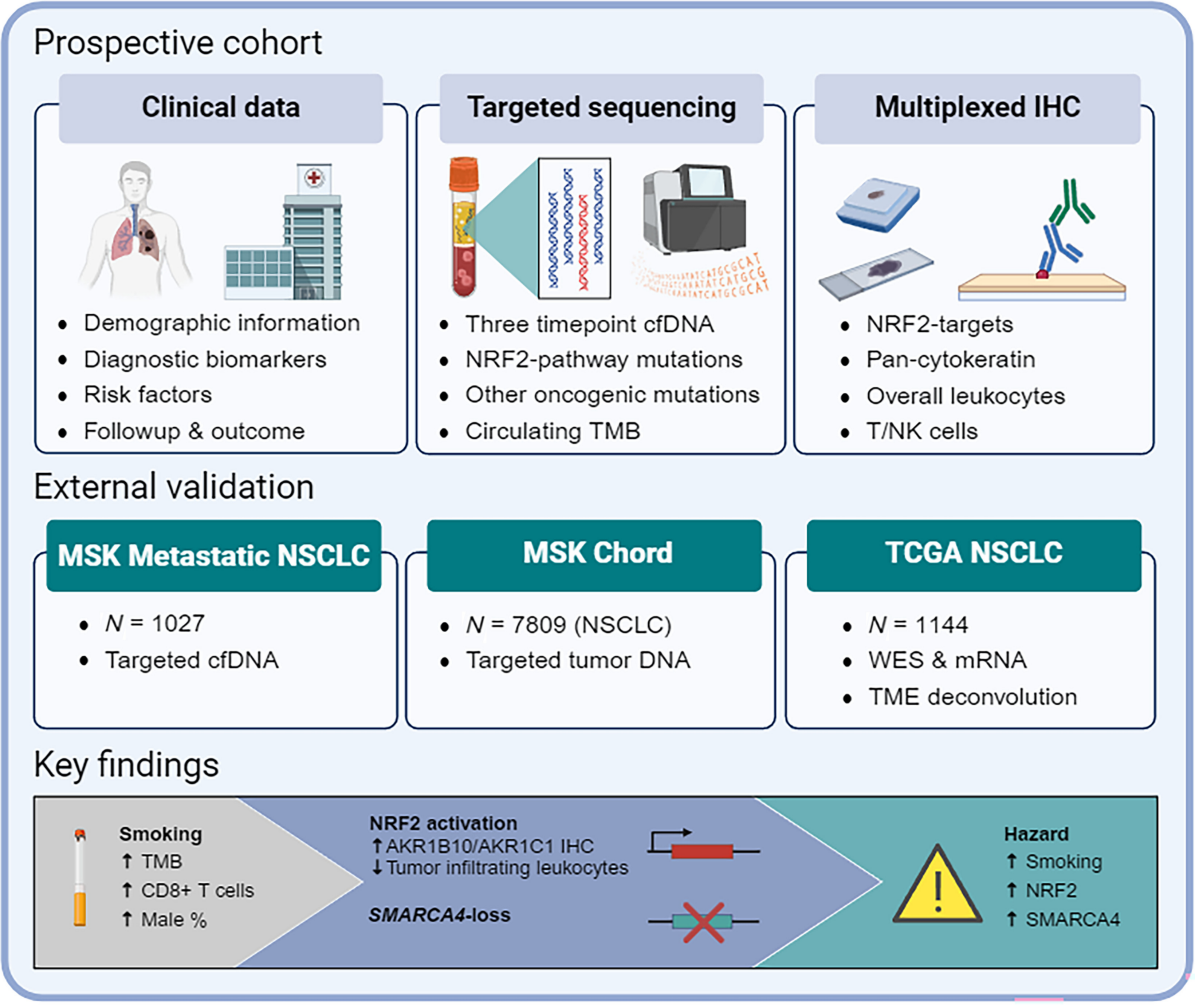
Overview of the study cohorts and key findings. cfDNA, cell‐free DNA; NRF2, nuclear factor erythroid 2‐related factor 2; NSCLC, non‐small cell lung cancer; TMB, tumor mutation burden; TME, tumor microenvironment. Created with BioRender.com.

In this study, we comprehensively characterized the clinicopathological associations of circulating pathogenic NRF2‐activating variants (Figure 4), showing a strong link to smoking and revealing cumulatively detrimental effects of NRF2 pathway and *SMARCA4* aberrations. While the correlations between *KEAP1* and *SMARCA4* as well as TMB have been established before [39], their combined effect on patient outcome has not, to our knowledge, been systematically evaluated in a prospective setting. Lung cancers in smokers are molecularly distinct from those in never smokers [40]. Importantly, most targetable receptor tyrosine kinase alterations (e.g., EGFR, ALK, ROS1) occur predominantly in never smokers, making individualized therapies disproportionately beneficial to this subgroup [40, 41, 42]. Moreover, given that NRF2 activation marks aggressive disease and remains untargetable, advocating smoking cessation is currently the only viable strategy to mitigate both its oncogenic effects and the broader burden of non‐targetable lung cancer. All analyzed datasets showed a higher incidence of NRF2 pathway and *SMARCA4* mutations in male patients, yet sex was not an independent predictor, suggesting that lower tobacco exposure accounts for the reduced incidence in female patients [43].

Our analyses indicated that *SMARCA4*‐mutated tumors exhibited enriched redox signaling and increased NRF2 target gene expression. Similarly, previous work shows that loss of *SMARCA4* selectively modulates NRF2‐driven genes, displaying directional variation. While HMOX‐1 showed systematic upregulation, some targets, including GCLC, were associated with more complex patterns, displaying bipotential regulation dependent on another SWI/SNF subunit, BRM, in addition to SMARCA4. Thus, modulation of SWI/SNF may result in further chemoresistance via selective dysregulation of NRF2 target genes, such as *HMOX1* [44]. This emphasizes the importance of selecting appropriate targets as biomarkers for NRF2 activation, as variance in some canonical downstream markers is partially explained by modulation of SWI/SNF function. These observations may explain our result on poor performance of GCLC as an NRF2 activity marker, contrasting to clearly concordant AKR1B10 and AKR1C1, further advocating for comprehensive screening of suitable biomarkers across conventional NRF2 targets. A study on pan‐cancer proteomics depicts AKR1B10 as one of the strongest components of the proteomic fingerprint of *NFE2L2*/*KEAP1*‐mutated cancers, further supporting our biomarker of choice and suggesting it is applicable to malignancies beyond NSCLC [45].

In our cohort, we observed one progression‐associated *KEAP1/SMARCA4* co‐mutation in a current smoker. To expand on mitigating risk factors driving NRF2 pathway activation during disease progression, future studies should evaluate the molecular consequences of smoking cessation from diagnosis through follow‐up in large prospective cohorts. In parallel, the high prevalence of oncogenic NRF2 activation in lung cancer (15–30%) and other malignancies underscores the urgent need for effective NRF2‐targeted therapies [13, 14, 15, 30]. Encouragingly, however, the decline in smoking rates may lead to a corresponding reduction in the incidence of NRF2‐hyperactive NSCLC. Moreover, various strategies to treat NRF2‐driven cancer are currently being explored [46, 47]. Some unsuccessful trials have been documented, including phase II studies with glutaminase inhibitor Telaglenastat and glutamine antagonist DRP‐104, terminated due to lack of benefit and strategic discontinuation, respectively [48, 49]. However, several phase I trials for direct NRF2 pathway perturbation are ongoing, including MGY825 and VVD130037 [50, 51]. Taken together, these efforts and developments depict promise towards improved clinical management of NSCLC.

This work also highlights that mutations in tumor suppressor genes such as *KEAP1* or *CUL3* may be ambivalent, as they are usually not restricted to specific loci. We demonstrated the utility of transcriptomic scoring of its target genes in a diagnostic setting, as well as the benefit of assessing target protein expression with IHC. For instance, *KEAP1* p.T609K and p.F190S were previously unknown variants that were confirmed NRF2 activating with IHC, and *KEAP1* p.K323A was confirmed as NRF2 activating with transcriptomic data, with all three cases presenting similar clinicopathological attributes to previously known NRF2 pathway hotspots. In addition, AKR1B10 expression was progressively associated with shorter survival as depicted in Figure 1D, and all cases harboring high AKR1B10 expression were deceased upon follow‐up. To further benchmark our IHC approach, we explored functional impact of rare variants with both molecular modeling and experimental mutagenesis reporter constructs, with all data leading to the same conclusions. Taken together, AKR1B10 IHC is a resource‐friendly solution to reveal functional pathway variants. Moreover, NRF2 activation is associated with chemotherapy resistance, and the stabilization of NRF2 may vary based on mutation [52, 53]. This warrants further investigation of the quantification of NRF2 activity levels across different NRF2 pathway aberrations in larger cohorts to unravel the full utility of these biomarkers. Collectively, this work supports the verification of rare variants of *KEAP1* and *CUL3* using IHC in diagnostic pathology, preferably employing markers AKR1B10 and AKR1C1 in conjunction. Moreover, the consistently poor outcomes associated with NRF2 activation across cohorts highlight the need to focus resources on this subgroup. To this end, accurate identification of bona fide NRF2 activation using complementary methods is essential to avoid false positives in diagnostics and research.

Our study explored the relationship between NRF2 activation and leukocyte infiltration, building on prior reports of reduced immune cell presence in NRF2‐activated tumors [30,54]. As our work expands on tumor immunity, we emphasize that while our study benefited from the prospective setting and is rich in observational data, it lacks mechanistic characterization of NRF2‐driven TME. To this end, during this work, other studies have explored the mechanistic role of NRF2 in its TME context, for which multiple explanations have been provided, and the field remains under active research. The most compelling evidence points towards multifactorial effects, including (a) dysfunctional priming of CD8+ T cells via inhibition of dendritic cell function [54]; (b) suppression of interferon‐induced genes via metabolic reprogramming and downregulation of STING/IFNα [55]; and (c) dysregulation of inducible programmed death‐ligand 1 (PD‐L1) via nicotinamide phosphoribosyltransferase [56]. In addition, our previous work suggests that Osteopontin (*SPP1*) is downstream of NRF2, and recently, it has been recognized as a potent immune‐checkpoint molecule [30,57]. Recent work shows that combination therapy of anti‐PD‐L1 improves outcomes in *KEAP1*/*STK11*‐mutated LUAD [58], suggesting that checkpoints beyond PD‐L1 may also contribute to NRF2‐associated immunological phenotypes. Collectively, pleiotropic anti‐inflammatory functions of NRF2 seem to result in the phenotypic observations in immune cell evasion spanning a wide set of leukocyte populations across different epithelial cancers, including NSCLC. Indeed, while most studies have shown a global shift towards an immune‐cold milieu, our observations suggest that high neoantigen load may offset NRF2‐driven immune suppression, particularly by preserving cytotoxic T‐cell infiltration [59]. Therefore, NRF2 mediated TME effects may not be inherently refractory to coexisting immunostimulatory cues. Given that the loss of KEAP1 impairs dendritic cell function (antigen presentation) and leads to reduced CD8+ T‐cell effector activity in murine models [54], the impact of neoantigens on CD8+ T‐cell cytolytic activity in NRF2‐activated tumors warrants further investigation. Interestingly, TCGA data show that the TMB effect is more evident in LUAD, while the immunological milieu of LUSC may be less affected by mutational content. In addition to the different compositions in cytotoxic T cells, lower mutational burden was associated with increased NK cells and more M2 type macrophages in LUAD, all of which align with lower HLA‐stimulated immunity. These effects were not observed in squamous lung cancer. Given that squamous metaplasia of the respiratory epithelium is a common outcome of extensive smoking and is inherently associated with increased genotoxic insults, early immunoediting may be more characteristic of squamous lung tumors, potentially explaining these findings. This difference might also clarify our previous observation that lower T‐cell populations were associated with NRF2 activation in LUSC, whereas a similar association was not seen in LUAD, possibly due to additional confounding factors. Notably, the present work comprises mainly late‐stage NSCLC of various subtypes, representing a clear demographic contrast to our earlier cohort of resected cases stratified by histology. In our cohort, features of NRF2‐activated disease (active smoking history, male sex, and the TMB–TME association) were observed across all subtypes, whereas in TCGA, these features were characteristic only of LUAD. This discrepancy may reflect differences in cohort composition, as our study included late‐stage cases, while TCGA underrepresents advanced NSCLC, likely due to limited availability of resected tissue in these cases. In addition, *KEAP1* and *NFE2L2* harbor differences in frequencies in NSCLC subtypes. In LUAD, *KEAP1* is more common, in contrast to LUSC where *NFE2L2* mutations are more frequent [60, 61]. Furthermore, beyond *SMARCA4*, *KRAS* and *STK11* also associate with *KEAP1* mutations in LUAD, and *STK11* mutations independently associate with an immunologically adverse TME. In these cases, combination therapy with anti‐PD‐L1 and anti‐CTLA4 resulted in clinical benefit [58]. In contrast to LUAD, in LUSC, NRF2 pathway aberrations are associated with *CDKN2A* mutations and *SOX2* amplification [30]. In our study, *KEAP1* and *NFE2L2* mutations were present in both subtypes, while the limited sample size restricted further analyses by therapeutic strategy, subtype, or mutation combination. These limitations underscore the need to further investigate NRF2 and SWI/SNF mutations, neoantigen load, and immune responses across disease stages and co‐occurring mutation combinations. Our study demonstrates that ctDNA‐based profiling combined with mIHC enables detailed molecular and immunological analysis from limited biopsy material, when surgical resection is not feasible.

While PD‐L1 remains the standard diagnostic marker for predicting ICB efficacy, TMB and CD8+ T‐cell infiltration have also shown predictive value [62, 63]. Our finding that NRF2 activation correlates with TMB highlights the need to further explore ICB efficacy in NRF2‐mutated cases, especially since TMB may influence the TME independently of NRF2. Recent studies have established the prognostic effect of NRF2 pathway activation across treatments, including ICB [58]. This underscores the limited efficacy of current therapies in NRF2‐hyperactive tumors and their role in driving aggressive NSCLC progression. Nonetheless, retrospective clinical data suggest that patients with NRF2‐activated tumors may still derive greater benefit from ICB compared with standard chemotherapy [64, 65]. Given the conflicting findings [54], future studies should address confounding factors, including treatment modality, lineage, PD‐L1 expression, CD8+ infiltration, and TMB, to comprehensively delineate the therapeutic benefit. To support accurate stratification in clinical studies of oncogenic NRF2 signaling, we recommend assessing somatic NRF2 pathway mutations alongside transcriptomic or protein‐level expression of established NRF2 targets to confirm both the presence and extent of bona fide pathway activation.

## Limitations

Although we have validated the main findings of this work with publicly available clinical cohorts, this study has limitations. Due to the scarcity of the core‐needle biopsies, the IHC findings do not necessarily represent the full heterogeneity of the tumors. This reflects the reality of diagnostic pathology in lung cancer, as biopsy material often falls short for comprehensive characterization. In addition, there was not enough tissue material to characterize matching mutations or TMB from the primary tumors, and as such, the sensitivity and specificity, as well as the clonal origin of the mutations derived from ctDNA could not be assessed by gold‐standard methodology. However, the following statements support the validity of our findings: (a) detected NRF2 pathway mutations matched with primary tumor IHC NRF2 targets; (b) bTMB aligned with smoking status (surrogate metric) and has been previously shown to correlate with TMB [37]; and (c) the top‐mutated genes aligned well with known NSCLC mutational profiles. Our prospective cohort represents unresectable cases that comprise mostly advanced stage disease, and as such, results should be interpreted accordingly.

Finally, our cohort included only one ICB‐treated case with a KEAP1 mutation, which was lost to follow‐up; therefore, we could not assess the combinatorial effect of ICB and NRF2 pathway alterations. The number of ICB‐treated AKR1B10‐positive cases was low (*n* = 5), and therefore we did not evaluate ICB response within this subgroup. However, in the full cohort, our Cox regression analyses indicate that AKR1B10 expression and ICB treatment have independent prognostic associations. We do not make therapeutic claims based on our data.

## Author contributions statement

JH, ST, PP and A‐LL conceived the study. PP and A‐LL supervised the study. Methodology was developed by JH, JK, TT, IP, PM, JPV and A‐LL. Software development was carried out by JH, TT and LM. Validation was performed by JH. Formal analysis was conducted by JH, TT and PM. Investigation was undertaken by JH, ST, LM, TT, IP, SA, TR and TP. Resources were provided by AM, PA, JPV and A‐LL. Data curation was performed by JH, ST, TT, LM and TR. The original draft of the manuscript was written by JH and A‐LL. Manuscript review and editing was completed by JK, TR, AM, TT, PM, IP, PA, JPV and PP. Visualization was carried out by JH, TT and PM. Project administration was managed by A‐LL. Funding acquisition was undertaken by JH, ST, PA and A‐LL.

## Supporting information


**Figure S1.** Circulating cell‐free DNA (cfDNA) metrics and biomarker benchmarking
**Figure S2**. Functional validation of *KEAP1* mutations
**Figure S3**. AKR1B10 proportional hazards models
**Figure S4**. Mutation burden metrics and other genomic context in non‐small cell lung cancer (NSCLC)
**Figure S5**. Circulating mutation dynamics, nuclear factor erythroid 2‐related factor 2 (NRF2)–SMARCA4 signaling and proliferation
**Figure S6**. Nuclear factor erythroid 2‐related factor 2 (NRF2) and cytotoxic lymphocytes in non‐small cell lung cancer (NSCLC)
**Table S1**. Clinical characteristics of the study cohort

**Table S2.** Genomic regions covered in the study gene panel (provided as a separate Excel file)

## Data Availability

Clinical and genomic data from the prospective cohort are not publicly available due to their sensitive classification in accordance with the General Data Protection Regulation (EU) 2016/679 (GDPR) and institutional ethical restrictions. Clinical and genomic datasets from the MSK cohorts are publicly available at https://www.cbioportal.org/. TCGA mRNA expression and mutation data were obtained from the Pan‐Cancer Atlas (https://gdc.cancer.gov/about‐data/publications/pancanatlas) (see Materials and methods). The data that support the findings of this study may be made available from the corresponding author on reasonable request and with the required institutional permissions.
