# Supplementary material for: Clinicopathological characteristics of patients with inoperable non‐small cell lung cancer harboring circulating NRF2 pathway mutations
Source: J Pathol. 2026 Mar 2;269(2):164–81. doi: 10.1002/path.70043 (PMC13140114; doi:10.1002/path.70043)
Supplement: Supplementary file 1 — Figure S1. Circulating cell‐free DNA (cfDNA) metrics and biomarker benchmarking Figure S2. Functional validation of KEAP1 mutations Figure S3. AKR1B10 proportional hazards models Figure S4. Mutation burden metrics and other genomic context in non‐small cell lung cancer (NSCLC) Figure S5. Circulating mutation dynamics, nuclear factor erythroid 2‐related factor 2 (NRF2)–SMARCA4 signaling and proliferation Figure S6. Nuclear factor erythroid 2‐related factor 2 (NRF2) and cytotoxic lymphocytes in non‐small cell lung cancer (NSCLC) Table S1. Clinical characteristics of the study cohort [file PATH-269-164-s002.docx]

**Clinicopathological characteristics of patients with inoperable non-small cell lung cancer harboring circulating NRF2 pathway mutations**

J Härkönen *et al. J Pathol* <https://doi.org/10.1002/path.70043>

**Supplementary Figures S1–S6**

**Supplementary Table S1**

**Supplementary Table S2 is provided as a separate Excel file**


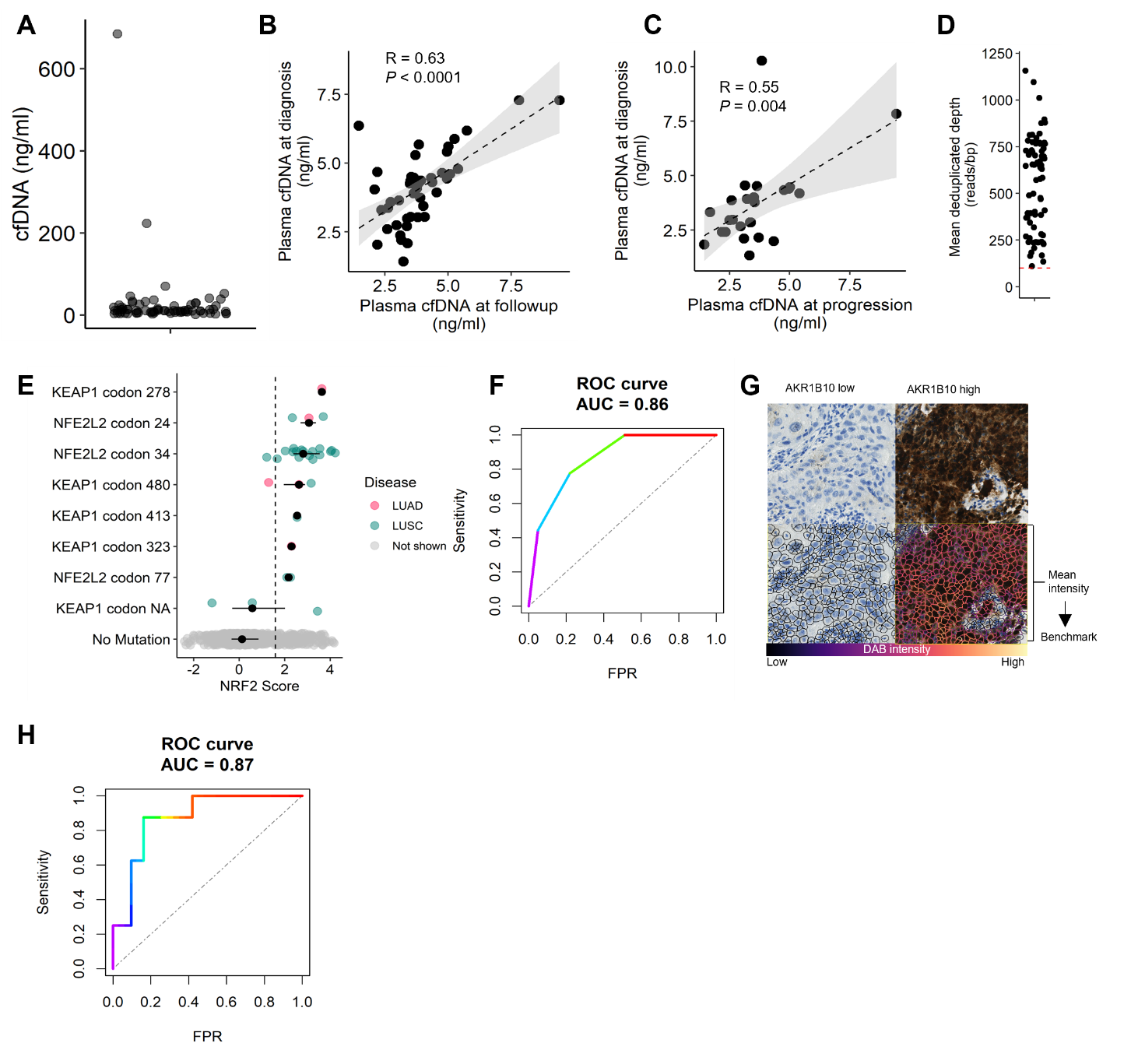


**Figure S1. Circulating cell-free DNA (cfDNA) metrics and biomarker benchmarking.** (A) Jitter plot of cfDNA concentrations in all cases at the time of diagnosis. (B) Scatter plot and correlation coefficient of cfDNA concentration at the time of diagnosis versus follow-up. (C) Scatter plot and correlation coefficient of cfDNA concentration at the time of diagnosis versus progression. (D) Deduplicated read depth in successfully sequenced cases. (E) NRF2 activity scores for all the overlapping codons in our study in TCGA data. (F) ROC-curve for human assessed AKR1B10 protein expression with immunohistochemistry. (G) Example patches from digital mean cellular intensity measurements in AKR1B10 high and low cases. Average expression was computed for concordance assessment. (H) ROC-curve for digitally calculated AKR1B10 expression versus NRF2 pathway mutations.


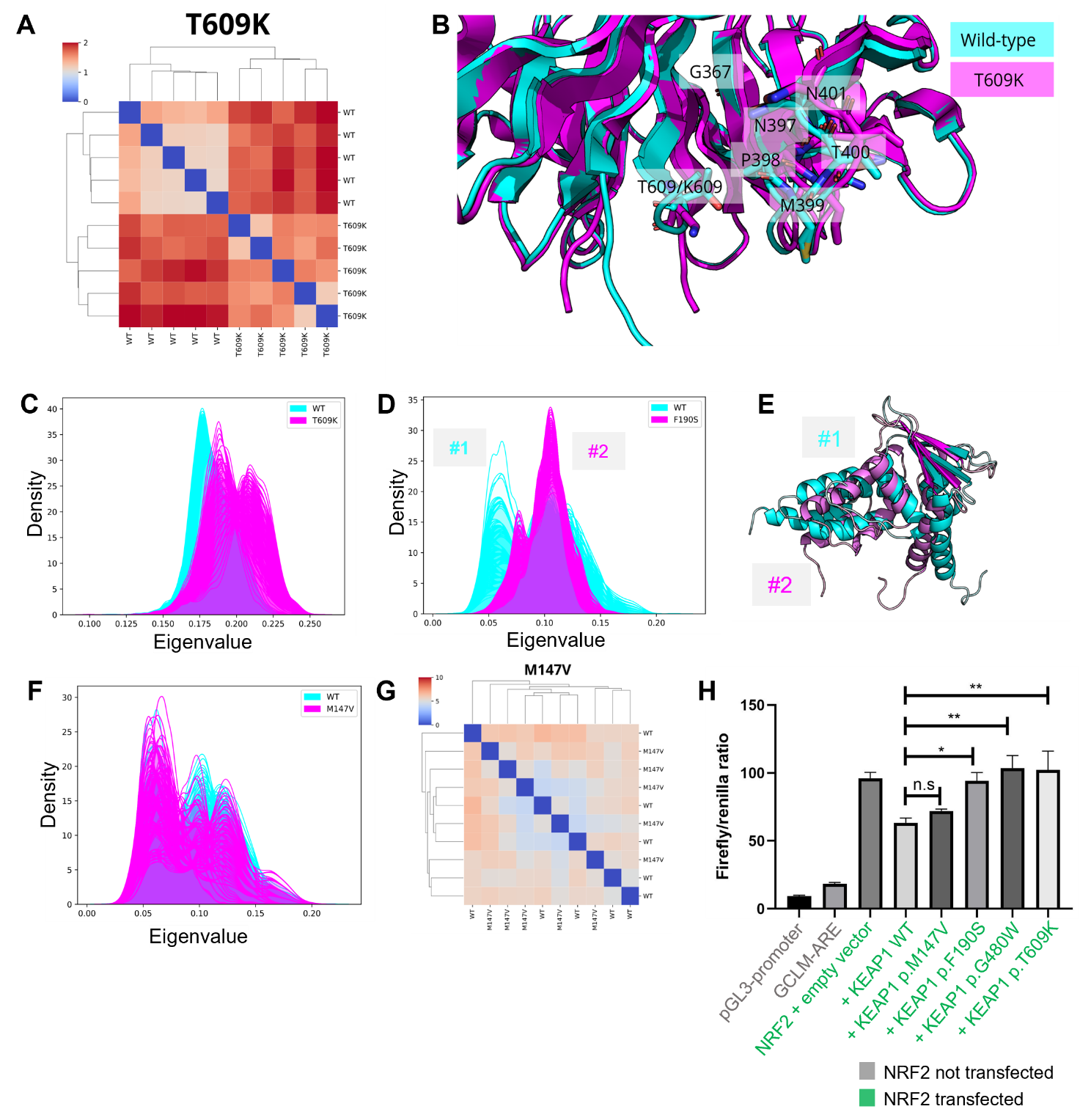


**Figure S2. Functional validation of *KEAP1* mutations.** (A) Cluster maps of directed Hausdorff distance matrices containing wild-type and mutated receptor molecular dynamics (MD) trajectories for *KEAP1* p.T609K. (B) Structural comparison on *KEAP1* p.T609K and wild-type structures, a nearby loop is forced to move away from the mutated residue, possibly due to larger size of the lysine residue compared to that of threonine. (C) Bootstrapped eigenvalue distributions of the MD trajectories from wild-type (cyan) and mutated (magenta) receptors. There is a slight shift in the eigenvalue distribution with T609K, consistent with the loop shift seen from visual evaluation of the averaged structures. (D) Bootstrapped eigenvalue distributions of the MD trajectories from KEAP1 wild-type (cyan) and p.F190S (magenta) receptors. The conformation steers from a bimodal distribution to unimodal. (E) Depicted dominant conformations of KEAP1 wild-type versus p.F190S. (F) Bootstrapped eigenvalue distributions of the MD trajectories from KEAP1 wild-type (cyan) and p.M147V (magenta), showing similar distributions. (G) Cluster maps of directed Hausdorff distance matrices containing wild-type and mutated receptor MD trajectories for KEAP1 p.M147V. (H) Firefly/Renilla luciferase activity in cells transfected with the indicated constructs. Green indicates co-transfection with the NRF2 expression construct.


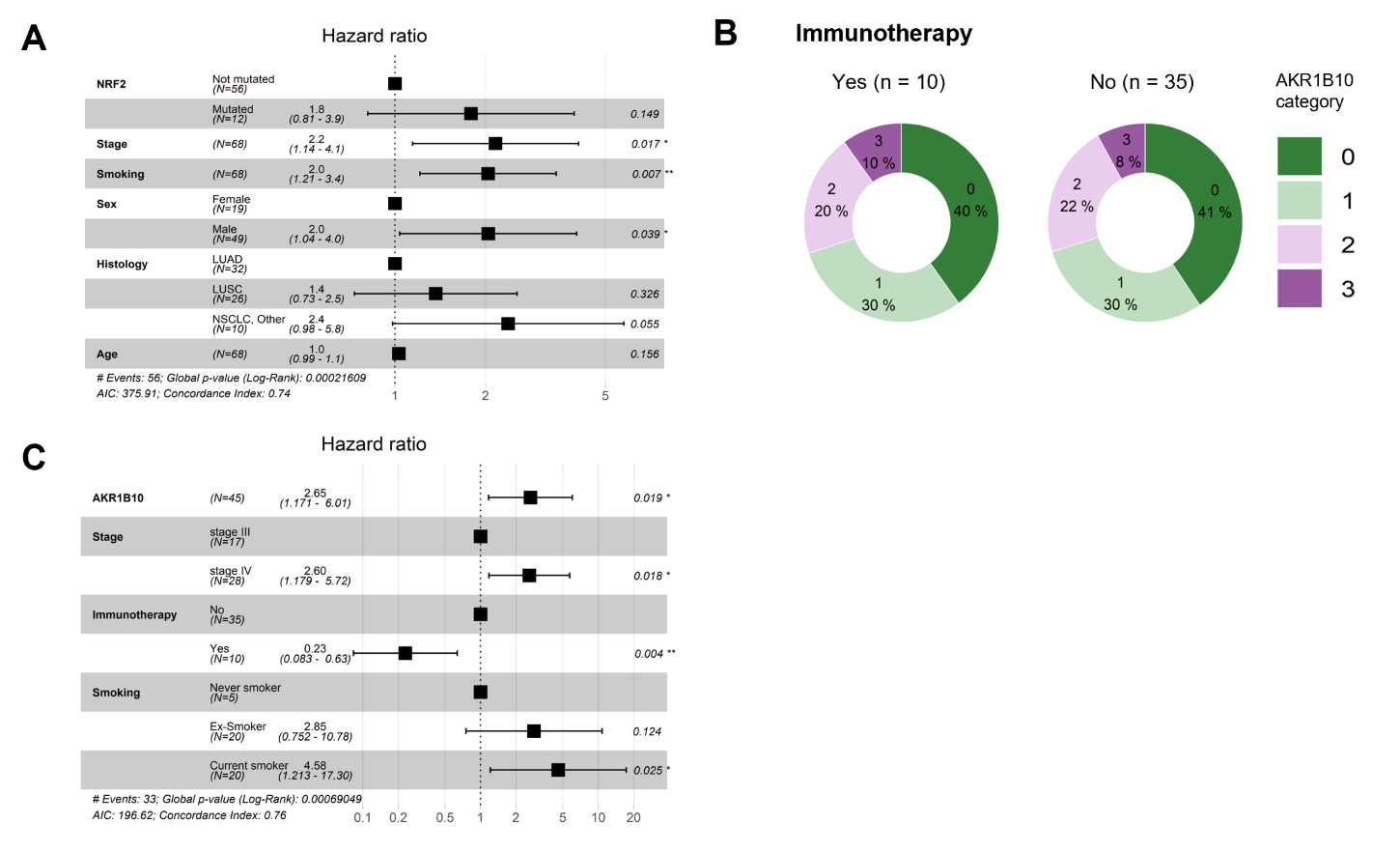


**Figure S3. AKR1B10 proportional hazards models. (**A) Hazard ratios and 95% CIs for disease progression from multivariable cox-regression with NRF2 pathway mutations, stage, smoking, sex, histology and age as predictors. (B) Percentages of categorical AKR1B10 expression in ICB-treated versus other cases. (C) Hazard ratios and 95% CIs for overall survival from multivariable cox-regression with AKR1B10 expression (< 2 versus ≥ 2), stage, immunotherapy and smoking as predictors.


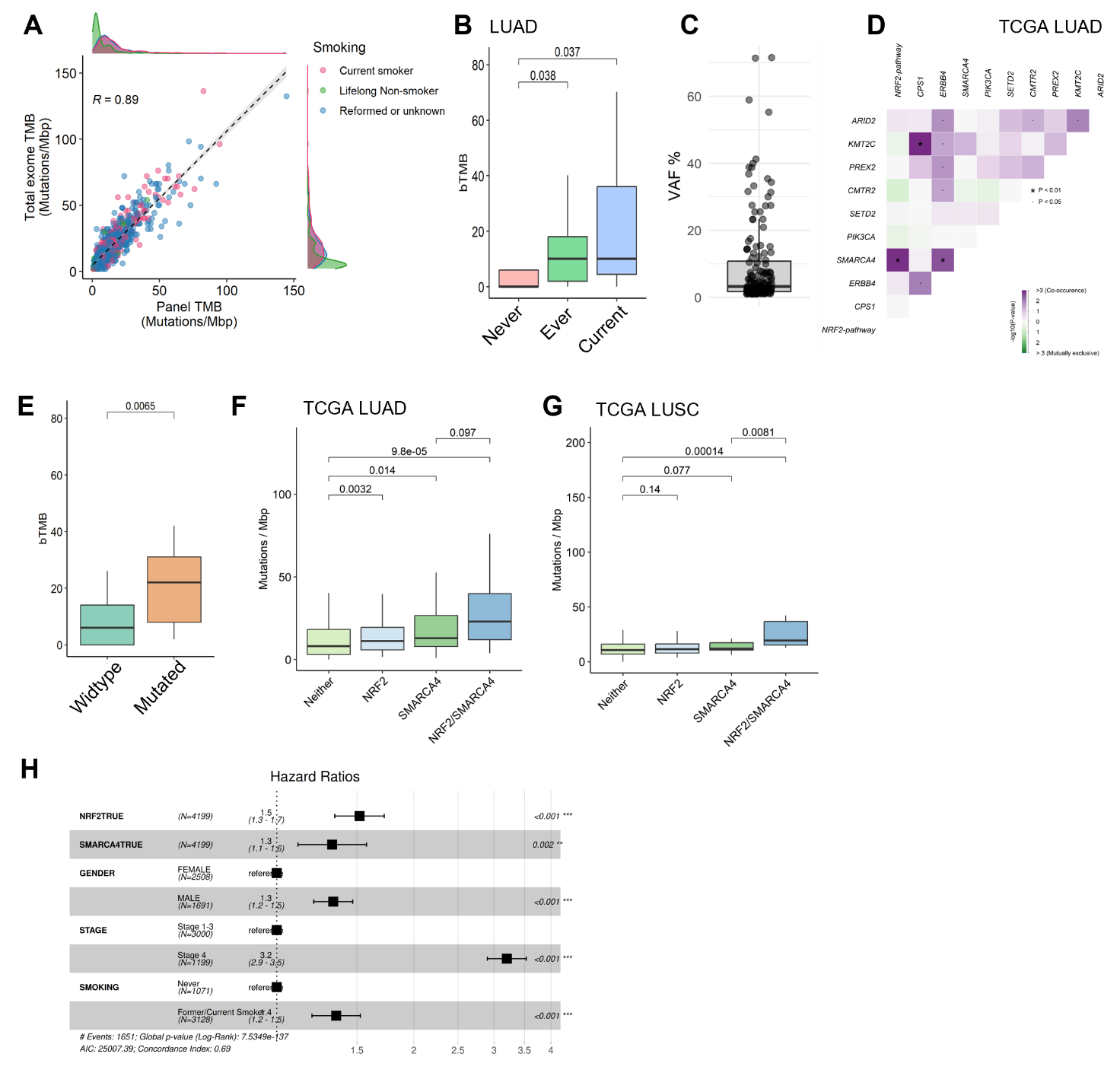


**Figure S4. Mutation burden metrics and other genomic context in NSCLC.** (A) Scatter plot and Pearson’s correlation coefficient for tumor mutational burden assessed from the panel-covered region versus whole-exome. (B) The association between smoking and circulating tumor mutational burden in lung adenocarcinoma (LUAD). (C) variant allele frequency (VAF) distribution of the pathogenic variants in our cohort. ≥ 1% was used as a hard threshold for minimum VAF. (D) Heatmap of Fisher’s exact test odds and *p*-values for somatic interactions in TCGA LUAD. (E) Blood-based tumor mutational burden (bTMB) in NRF2 pathway mutated and wildtype samples. (F) Tumor mutational burden in the NRF2 pathway, *SMARCA4* and co-mutated cases in TCGA-LUAD. (G) Tumor mutational burden in the NRF2 pathway, *SMARCA4* and co-mutated cases in TCGA-LUSC. (H) Hazard ratios and 95% CIs for overall survival from multivariable cox-regression with NRF2 pathway and *SMARCA4* mutations, stage, sex and smoking as predictors.


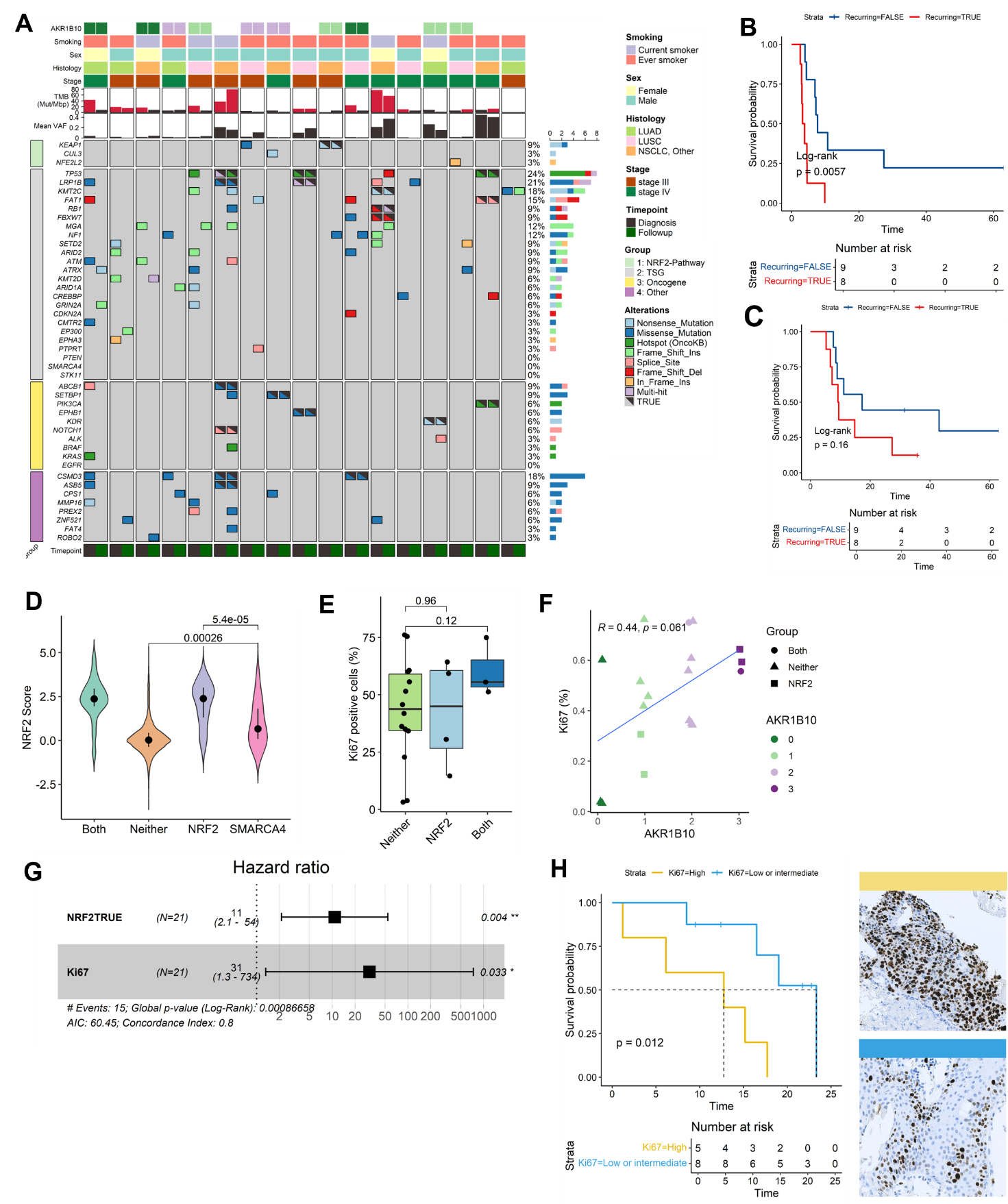


**Figure S5. Circulating mutation dynamics, NRF2–SMARCA4 signaling and proliferation.** (A) Mutations in circulating tumor DNA (ctDNA) in diagnosis and progression timepoints. Recurring alterations are marked with a black triangle. (B) Progression-free survival for non-recurring versus recurring circulating mutations depicted in light blue and blue, respectively. (C) Overall survival for non-recurring versus recurring circulating mutations depicted in light blue and blue, respectively. (D) NRF2 activity score comparison among cases with NRF2 pathway mutations, *SMARCA4* mutations, no mutations in either of the genes, and cases harboring both mutations. (E) Ki67-proliferation index in NRF2 and *SMARCA4*/NRF2-mutated cases. (F) Ki67 proliferation index across categorized AKR1B10 expression. (G) Cox multivariable regression hazard ratios for Ki67 proliferation index and NRF2 pathway mutations. (H) Kapplan–Meier curve for high versus low Ki67 samples without NRF2 pathway mutations (left panel), and representative images of high versus low Ki67 samples (right panel).

**
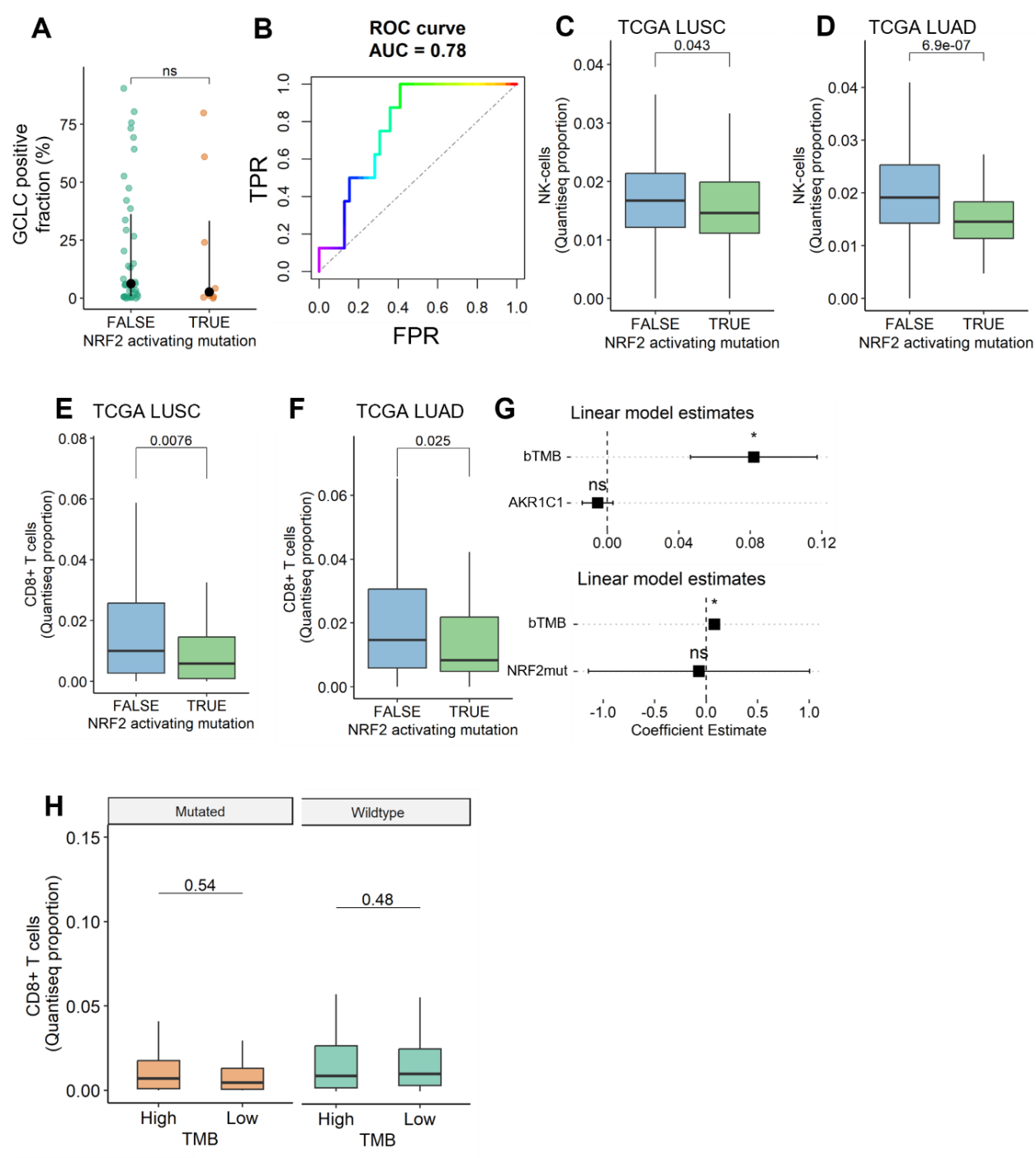
**

**Figure S6. NRF2 and cytotoxic lymphocytes in NSCLC.** (A) GCLC protein expression between NRF2 pathway mutated versus non-mutated cases. (B) Receiver operating characteristics (ROC)-curve for mean cancer cell AKR1C1 intensity versus NRF2 pathway mutation status. (C) Comparison of deconvoluted natural killer (NK) cells between NRF2 pathway mutated and non-mutated cases in TCGA-LUSC. (D) Comparison of deconvoluted NK cells between NRF2 pathway mutated and non-mutated cases in TCGA-LUAD. (E) Comparison of deconvoluted cytotoxic T cells between NRF2 pathway mutated and non-mutated cases in TCGA-LUSC. (F) Comparison of deconvoluted cytotoxic T cells between NRF2 pathway mutated and non-mutated cases in TCGA-LUAD. (G) Linear model predictors of CD8+ T cell density in NSCLC-tumors, including blood-based tumor mutational burden (bTMB) and AKR1C1 (upper panel) and bTMB and NRF2 pathway mutations (lower panel). (H) Deconvoluted cytotoxic T cell proportions between TMB-high and TMB-low cases in NRF2 mutated (green) and non-mutated (blue) groups in LUSC.

**Table S1.** Clinical characteristics of the study cohort.

| **Characteristic** | | **Total *n* (%)** |
| --- | --- | --- |
| All cases | | 73 (100%) |
| **Sex** | |  |
| Male | | 53 (73%) |
| Female | | 20 (27%) |
| **Age (yrs)** | |  |
| < 65 | | 18 (25%) |
| ≥ 65 | | 55 (75%) |
| **Histology**  Squamous cell cancer  Adenocarcinoma  NSCLC, NOS | | 29 (40%)  32 (44%)  12 (16%) |
| **AJCC stage** |  | |
| I–II | | 5 (7%) |
| III–IV | | 68 (93%) |
| **Smoking status**  Never smoker  Ex-smoker  Current smoker | | 11 (15%)  33 (45%)  29 (40%) |
| **PD-L1**  > 50% | | 13/57 (23%) |
| 1–49% | | 25/57 (44%) |
| < 1% | | 19/57 (33%) |
| NA | | 16/73 (22%) |
| **Immunotherapy**  Yes  No  NA | | 13/69 (19%)  58/73 (81%)  1/73 (1,4%) |
| ***EGFR* mutation in tumor** (Idylla™) | | 7/44 (16%) |

AJCC, American Joint Committee on Cancer.
